# Smartphone-Based Field Assessment of Trunk Stability and Its Relationship With Whole-Body Balance in Older Adults: Cross-Sectional Study

**DOI:** 10.2196/83546

**Published:** 2026-05-06

**Authors:** Javier De Los Ríos-Calonge, Casto Juan-Recio, Amaya Prat-Luri, Aarón Miralles-Iborra, Jose LL Elvira, David Barbado, Francisco Jose Vera-Garcia

**Affiliations:** 1 Sports Research Centre, Department of Sport Sciences, Miguel Hernández University of Elche, Avda. de la Universidad s/n., Elche, 03202, Spain, 34 692102969; 2 Alicante Institute for Health and Biomedical Research (ISABIAL), Alicante, Spain

**Keywords:** mHealth, smartphone accelerometry, field tests, association, core stability, postural balance, screening, aging

## Abstract

**Background:**

Maintaining balance is essential for older adults to preserve independence and reduce fall risk. However, empirical evidence linking trunk stability with balance and gait is scarce, partly due to the lack of accessible field tests. Smartphone-based accelerometry field tests offer a promising approach to assess trunk stability outside the laboratory.

**Objective:**

The study aimed to determine (1) the association between trunk stability and whole-body balance and gait and (2) the extent to which trunk stability and whole-body balance field tests align with laboratory-based assessments in older adults.

**Methods:**

A total of 53 physically active older adults performed 2 trunk stability tests (ie, the lumbopelvic stability [field] and the unstable sitting posturographic [laboratory] tests), 2 whole-body balance tests (ie, the tandem stance posturographic [laboratory] and the whole-body static balance [field] tests), and 2 gait-related tests (ie, the straight-line gait and the modified Timed Up and Go [field] tests) 2 times. A Spearman correlation analysis was performed after assessing the reliability of the variables.

**Results:**

Significant positive low-to-high correlations were found between the lumbopelvic stability and the whole-body static balance tests (0.380≤*r*≤0.700; *P*<.01). A low positive correlation (*r*=0.436; *P*<.01) was also observed between the unstable sitting posturographic test and the tandem stance posturographic test. No correlations were found between trunk stability and any gait-related test. No moderate-to-high relationships (0.289≤*r*≤0.487; *P*<.05) were found between laboratory and field tests (both for trunk stability and whole-body balance).

**Conclusions:**

Better static lumbopelvic control during exercises like the back bridge and the bird-dog is linked to improved whole-body balance in active older adults, suggesting that these positions be included in future interventions to enhance trunk stability. Accessible smartphone-based field tests for lumbopelvic stability offer a promising approach for the standardized assessment of trunk stability. However, direct extrapolation from field tools to laboratory findings is unfeasible due to a lack of correlation. These findings highlight the potential of smartphone-based field tests to complement clinical and research assessments of balance in older adults.

## Introduction

Maintaining balance and gait in older adults is crucial for functional independence and reducing fall risk [1]. Biomechanical research suggests that proper trunk neuromuscular control could play a key role in preserving motor function in this population, as it has been linked with the ability to maintain an upright posture [2], transfer loads [3], and coordinate movements during daily activities [4]. Previous studies that measured trunk stability (ie, understood as the capability to maintain or resume a relative position or trajectory of the trunk following internal or external forces) [5] in both laboratory [6] and field settings [7] support this idea, as they found reduced voluntary trunk control among older people in seated positions compared to young individuals [6], and they were able to discriminate between fallers and nonfallers [7]. More recent research has used smartphone-based accelerometry at the thoracic level to examine associations between trunk accelerations during upright balance and functional tasks, such as the sit-to-stand task, in older adults [8-10]. While these approaches provide valuable insights into higher global postural control during standing tasks, trunk behavior is evaluated within movements in which control is distributed across multiple joints and segments. Consequently, there is a lack of empirical evidence directly linking trunk-focused stability outcomes (ie, derived from tasks designed to impose relatively high demands on lumbopelvic control) with whole-body balance and gait-related parameters in older adults. In this sense, while trunk-focused exercise programs have proven to be useful to improve balance and gait in this population [11], it remains unclear to what extent these benefits stem from enhanced trunk stability, as this capability is not typically assessed. Moreover, although correlational studies have shown positive relationships of trunk muscle strength [12-14], endurance [13], muscle thickness [15,16], and spinopelvic range of motion [12,17,18] with whole-body balance (ie, static and dynamic) [12,18], gait parameters (ie, step length, gait speed) [14,17,18], and functional capacity (ie, the time taken to complete the sit-to-stand and the Timed Up and Go tests) [12,14], correlational evidence specifically addressing trunk stability remains scarce. To the authors’ knowledge, only 2 studies have linked trunk-focused stability outcomes from a seated dynamic postural control test on a force platform (in which lower-limb and arm strategies are largely constrained, making task performance predominantly dependent on trunk control) with observational-based assessments of whole-body balance and gait in older adults [19,20].

A key reason for the limited research in this field is the absence of field tests that objectively quantify trunk stability. Inertial sensors have emerged as cost-effective field-setting tools to reliably detect postural control impairments in older adults [21]. While laboratory-based force platform assessments remain the standard reference for biomechanical analysis, their limited accessibility has motivated the development of field-based alternatives. Based on this approach, our research group has developed a novel accelerometry-based field method [22] to assess trunk-focused stability outcomes during common floor-based trunk exercises (eg, bridges and bird-dogs). While this method has shown to be promising in young adults [23], its applicability and correlation with whole-body balance and gait in older adults remain unexplored. Since these exercises are used in exercise programs that aim to preserve motor function in older people [24,25], introducing an accelerometry-based assessment of trunk stability would help to establish which exercises are most closely associated with whole-body balance and gait and thus should be prioritized within training programs.

Given this scarcity of direct empirical evidence, the primary aim of this study was to explore the associations between biomechanically based trunk stability, whole-body balance, and gait outcomes in physically active older adults. This was managed by using both well-established laboratory protocols and novel inertial-sensor-based field tests: (1) trunk stability tests: the unstable sitting posturographic test (laboratory-based) [5] and the lumbopelvic stability tests (field-based) [22]; (2) whole-body balance tests: the tandem stance dynamic posturographic test (laboratory-based) [26] and the whole-body static balance test (field-based) [27]; and (3) gait-related tests: the straight-line gait [27] and the modified Timed Up and Go (field-based) [28] tests. A secondary exploratory aim was to analyze the relationships between these laboratory and field assessments to inform practitioners about their alignment. Finally, this study aimed to determine the between-session test-retest reliability (both relative and absolute) of all outcomes, to support interpretation of the correlational findings, and to guide the selection of robust and reproducible performance metrics. Based on the principle of specificity and on previous studies [23,26], it was first hypothesized that trunk stability would show moderate associations with biomechanical whole-body balance outcomes only in those tests that share task requirements. Specifically, we hypothesized that lumbopelvic stability tests would correlate with whole-body static balance tests, given the shared demand for maintaining steady positions (eg, standing, bridging, and bird-dog). Thereafter, we hypothesized that the unstable sitting posturographic test would correlate with the tandem stance posturographic test due to their common requirement to anticipate and respond to predicted disturbances. Regarding gait-related outcomes, we hypothesized that there would be no correlation with trunk stability tests, except between the unstable sitting posturographic test and the modified Timed Up and Go test, since voluntary trunk control may play a key role in standing, sitting, and turning.

## Methods

### Participants

In order to check the proposed hypotheses, a correlation coefficient (*r*) of 0.5 was expected for the sample size estimation, considering previous studies that examined the relationship between trunk stability tests and whole-body dynamic balance tests [23,26]. The software G*Power 3.1 (v3.1, University of Düsseldorf) was used with the following parameters: *r*=0.5; *α*=.006; 1*–β*=.8. Calculations suggested a sample size of approximately 46 participants. An additional 20% of the target sample was recruited to anticipate the loss of subjects due to the inability to implement some of the tests presented. A total of 53 volunteer participants aged 65 years or older (mean age 70.3, SD 3.8 y; 21 male participants and 32 female participants) were recruited for the study. The main sociodemographic and anthropometric characteristics and the physical activity levels of the participants are shown in Table 1. Moreover, Table S1 in Multimedia Appendix 1 shows the clinical data of the participants. Participants were asked to present a medical certificate signed by their primary care physician to guarantee that they had no contraindications to perform trunk stability, whole-body balance, and gait-related tests.

**Table 1.** Main sociodemographic and anthropometric characteristics and physical activity levels of the participants.

	Male participants (n=21)	Female participants (n=32)	Total (N=53)
Age and anthropometric data, mean (SD)			
Age (y)	70.3 (4.1)	70.3 (3.7)	70.3 (3.8)
Body mass (kg)	77.1 (9.0)	64.6 (7.9)^a^	69.6 (10.3)
Height (m)	1.70 (0.07)	1.56 (0.06)^a^	1.62 (0.09)
Trunk height (m)	0.57 (0.03)	0.50 (0.04)^a^	0.53 (0.05)
Body moment of inertia (kg·m^2^)	67.6 (10.3)	47.7 (7.0)^a^	55.6 (12.9)
Trunk moment of inertia (kg·m^2^)	9.8 (1.7)	6.5 (1.5)^a^	7.8 (2.3)
Sociodemographic data, n (%)			
Education			
Primary studies	3 (14)	12 (38)	15 (28)
Secondary studies	12 (57)	12 (38)	24 (45)
Higher studies	3 (14)	2 (6)	5 (9)
Vocational training	0 (0)	5 (16)	5 (9)
None	3 (14)	1 (3)	4 (8)
Living alone	0 (0)	8 (25)	8 (15)
Financial difficulties	1 (5)	3 (9)	4 (8)
Physical activity data, n (%)			
Physically active for the past 20 y			
>150 mins/wk	10 (48)	15 (47)	25 (47)
≤150 mins/wk	11 (52)	17 (53)	28 (53)
Performing strength training	4 (19)	15 (47)	19 (36)
Currently physically active			
>150 mins/wk	14 (67)	20 (63)	34 (64)
≤150 mins/wk	7 (33)	12 (38)	19 (36)
Performing strength training	9 (43)	21 (66)	30 (57)

a Pairwise comparisons between sexes: significant differences (*P*<.05).

### Ethical Considerations

The University Office for Research Ethics (DCD.FBM.01.23) approved this study. The study was conducted in accordance with the Declaration of Helsinki, informing the participants of the characteristics and risks of the study. Each participant provided written informed consent. Data were handled to ensure privacy and confidentiality and were accessible only to the research team. No financial compensation was provided. Written informed consent was obtained for the publication of the participant’s images.

### Experimental Procedure

Each participant’s mass (mean 69.6, SD 10.3 kg); height (mean 1.62, SD 0.10 m); and height of the center of mass position of the head, arms, and trunk segment (estimated at 62.6% [29] of the trunk height, measured as the distance between the greater trochanter and the glenohumeral joint: 0.53, SD 0.05 m) were registered before testing. The participant’s preferred leg was established as the leg that the participant would use to kick a ball. Participants carried out 2 testing sessions with a day’s rest in-between, and these sessions were repeated 1 week later. The first assessment session consisted of a trunk stability assessment through lumbopelvic stability tests (using a randomized block design to avoid fatigue influence) during the back bridge, the preferred side bridge (determined by the side of the preferred leg), and the front bridge and the bird-dog positions [22]. The second assessment session consisted of trunk stability, dynamic balance, static balance, and gait-related tests (carrying out first those tests in which performance is most likely to be affected by fatigue) performed in the following order: (A) the unstable sitting posturographic test, (B) the tandem dynamic balance stance posturographic test, (C) whole-body static balance tests, (D) straight-line gait tests*,* and (E) the modified Timed Up and Go test. A 5‐ to 7-minute warm-up was performed at the beginning of each testing session to enhance awareness of spine and pelvis alignment and to facilitate the understanding and practice of neutral spine posture. The following exercises were included in this warm-up: (1) isolated scapular retractions and protractions; (2) isolated pelvic retroversions and anteversions; and (3) the cat-camel exercise.

### Testing Protocol Description and Data Processing

#### Trunk Stability Tests

**Figure 1.**
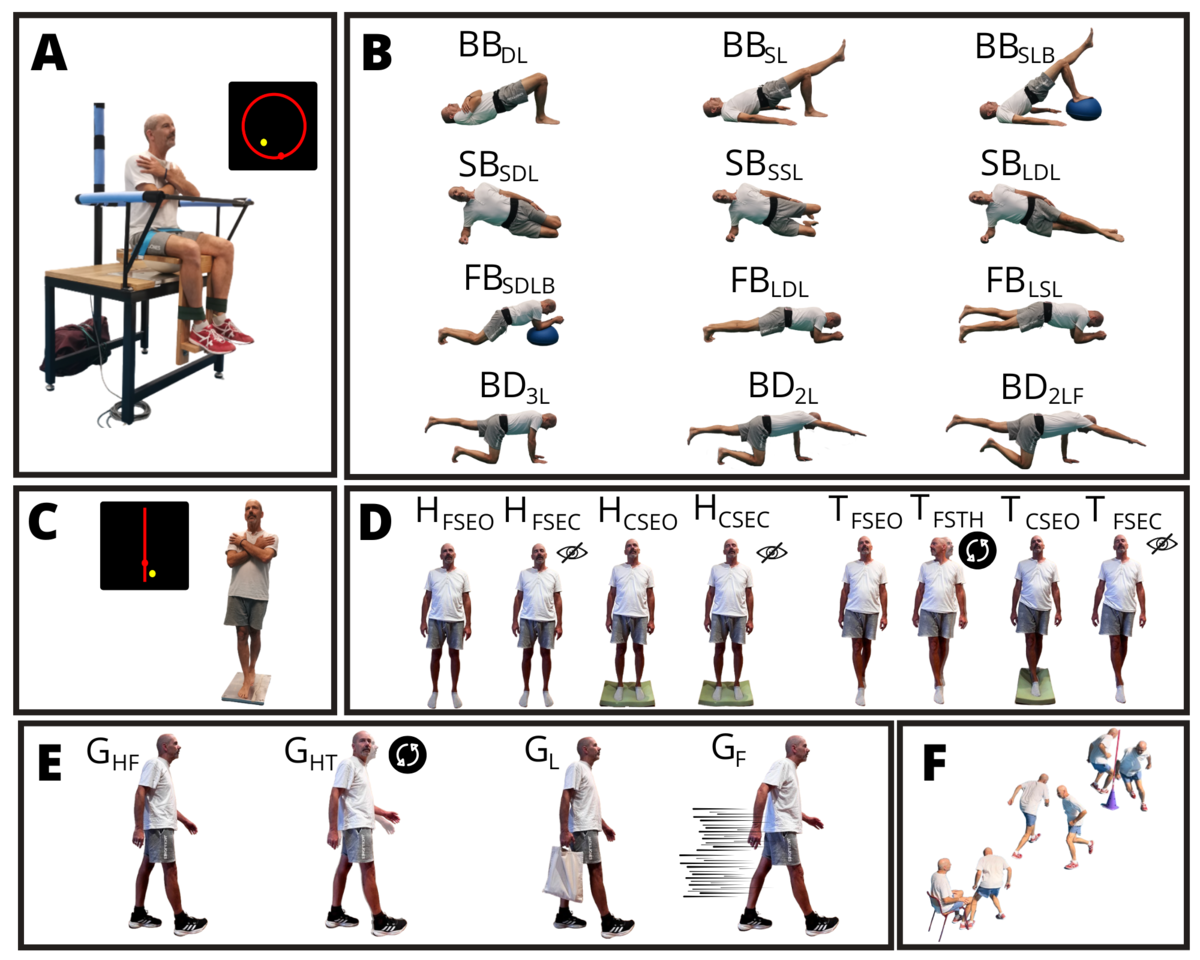
Trunk stability, whole-body balance, and gait-related tests: (A) The unstable sitting posturographic test. (B) The lumbopelvic stability tests performing 3 back bridge (back bridge with double-leg support [BB_DL_], back bridge with single-leg support [BB_SL_], and back bridge with single-leg support on a hemisphere ball [BB_SLB_]), 3 preferred side bridge (side bridge with short double-leg support [SB_SDL_], side bridge with short single-leg support [SB_SSL_], and side bridge with long double-leg support [SB_LDL_]), 3 front bridge (front bridge with short double-leg support and arms on hemisphere ball [FB_SDLB_], front bridge with long double-leg support [FB_LDL_], and front bridge with long single-leg support [FB_LSL_]), and bird-dog (3-limb bird-dog position [BD_3L_], 2-limb bird-dog position [BD_2L_], and 2-limb bird-dog position with the forefoot of the supporting leg elevated [BD_2LF_]) positions. (C) The tandem stance posturographic test. (D) The whole-body static balance tests performed with feet hip-width apart (firm surface and eyes open [H_FSEO_], firm surface and eyes closed [H_FSEC_], compliant surface and eyes open [H_CSEO_], and compliant surface and eyes closed [H_CSEC_]) and tandem (firm surface and eyes open [T_FSEO_], firm surface and turning the head [T_FSTH_], compliant surface and eyes open [T_CSEO_], and firm surface and eyes closed [T_FSEC_]) positions. (E) Straight-line gait tests (head-forward gait [G_HF_]; head turning gait [G_HT_]; 1-sided 10% body weight load gait [G_L_]; fast gait without running [G_F_]). (F) The modified Timed Up and Go test performed as fast as possible, being allowed to run. Written informed consent was obtained for the publication of the participant’s images.

Lumbopelvic linear accelerations were recorded to assess the postural control challenge imposed on the participants as an index of exercise intensity during the lumbopelvic stability tests (field-based), which involved performing three 15-second back bridge, preferred side bridge, front bridge, and bird-dog positions (Figure 1B) using a novel “ad hoc” smartphone app (CoreMaker) created by our research group. A smartphone was placed with Velcro above the participant’s sacrum on an elastic pelvic belt to reduce smartphone movement caused by muscle contractions similar to the protocol previously described by Heredia-Elvar et al [22]. The accelerations were recorded at 100 samples per second from a 3-axis accelerometer embedded in the smartphone (iPhone SE, 2nd generation, model MHGQ3QL/A; Apple Inc.), and it was configured using iOS version 15. Lumbopelvic stability was assessed through the mean acceleration (m·s^-2^) calculated as the average of the acceleration magnitude data series obtained from the 3-axis (vertical, anterior-posterior, and medial-lateral) of the smartphone accelerometer using the CoreMaker app mentioned earlier.

The unstable sitting posturographic test (laboratory-based) [5] was used to assess the participants’ ability to control their trunks during a circular tracking task while sitting on an unstable seat placed on a force platform (9287CA, Kistler; Figure 1A). Real-time feedback of the participants’ center of pressure displacement was displayed on a screen. Additionally, a target point (which moved in a circular trajectory) was presented to assess the participants’ ability to adjust their center of pressure position to this point. A 4° inclination angle of the head, arms, and trunk center of mass (estimated at 62.6% [29] of the distance between the greater trochanter and the glenohumeral joint) was calculated to establish the movement amplitude of the target point. The participants’ arms were crossed over their chest, and they performed five 70-second trials with a 60-second rest between trials. Center of pressure displacement was collected and filtered by a low-pass-Butterworth filter with a cutoff frequency of 5 Hz. The initial 10 seconds were discarded due to the nonstationarity of the signal. The mean radial error was used to quantify the trunk performance, calculated as the average of the vector distance magnitude (mm) of the center of pressure from the target point using an “ad hoc” software developed by our research group through LabView 9.0 environment (v9.0, National Instruments) [23].

#### Whole-Body Dynamic Balance Test

The tandem stance posturographic test (laboratory-based; Figure 1C) was used to measure the whole-body dynamic balance through an anterior-posterior tracking task (with the same characteristics as those used in the unstable sitting posturographic test but with a linear tracking task) while standing on a force platform (9286AA, Kistler) [26]. A 2° inclination angle of the whole-body center of mass (estimated at 55% [29] of each participant’s height) was calculated to establish the movement amplitude of the target point. The participants performed the tandem test with their preferred lower limb placed ahead of the other limb. Their arms were crossed over their chest during both tests, and they performed each task 3 times with a 1-min rest between tasks, with a trial duration of 70 seconds. Similar to the unstable sitting posturographic test, center of pressure displacement was filtered (low-pass Butterworth filter; cutoff frequency: 5 Hz), and the initial 10 seconds were discarded [5]. Then, the mean radial error (mm) was calculated to quantify balance performance.

#### Whole-Body Static Balance Tests

The whole-body static balance tests (field-based) evaluated participants’ postural sway through the manipulation of sensory inputs required for balance [27]. Participants wore an elastic sacroiliac belt fastened with Velcro around the lower back (around L5/S1) [27], which housed a smartphone (iPhone SE, 2nd generation, model MHGQ3QL/A). Data from the smartphone inertial sensors (accelerometer) were recorded during the tasks by the G&B smartphone app [27] at 100 samples per second. Participants were asked to stand as still as possible with their feet hip-width apart (H) and in tandem (T; Figure 1D) positions with their arms by their sides. The hip-width stance posturographic-field tests under steady-state conditions included standing on a firm surface and eyes open, on a firm surface and eyes closed, on a compliant surface and eyes open, and on a compliant surface and eyes closed. The tandem stance posturographic-field tests under steady-state conditions consisted of standing on a firm surface and eyes open, on a firm surface while turning the head, on a compliant surface and eyes open, and on a firm surface and eyes closed. The total of 8 static balance tasks (Figure 1D) were performed twice. Each trial lasted 30 seconds. The 8 tasks were performed in the order shown in Figure 1 with a rest period of 30 seconds between tasks. After a 2-min resting period, the tasks were performed again in the same order. A medium density foam mat (52 kg/m^3^, 50×28×5 cm) was used as the compliant surface. The original postural stability outcome obtained from the G&B was inverse log transformed to compute the mean absolute acceleration in each balance task.

#### Straight-Line Gait Tests

Four gait tests in a straight line were proposed to assess walking balance (gait periodicity index) and walking speed (Figure 1E). These included head-forward gait, head turning gait, one-sided 10% body weight load gait, and fast gait without running. The gait tasks were designed to be performed at the user’s preferred walking speed, except for the fast gait without running. Each task consisted of four 6-second walks. This task design allows the gait assessment to be carried out over approximately 10 m. The 4 tasks were performed in the order shown in Figure 1 with a rest period of 30 seconds between tasks, and a second set was performed in the same order after a 2-minute rest. As in the whole-body static balance tests, participants wore an elastic sacroiliac belt fastened with Velcro around the lower back (around L5/S1) [27] to house a smartphone (iPhone SE, 2nd generation, model MHGQ3QL/A). Data from the smartphone inertial sensors (accelerometer and gyroscope) were recorded by the G&B app [27] at 100 samples per second during the tasks. Specifically, a wavelet-based step-event detection algorithm and a double-pendulum gait model were used to analyze gait data by obtaining walking balance (periodicity index, %) and walking speed (m/s) outcomes through the G&B app. All straight-line gait and whole-body static balance assessments administered using the G&B smartphone app were adapted from the protocol described by Rashid et al [27], introducing additional task conditions to increase relevant task-specific postural and locomotor demands for older adults. These conditions were designed to progressively perturb balance control pathways, thereby increasing demands on segmental postural control, including the trunk.

#### Functional Mobility Test

The modified Timed Up and Go test consisted of standing up from a chair, moving forward 3 m, turning around a cone, returning to the chair, and sitting back down [28] (Figure 1F). Participants performed the task as fast as possible, including running if they were able, to better discriminate between participants and to avoid ceiling effects, particularly in active populations. The time (s) was recorded using a digital chronometer (HS-30W-N1V, CASIO).

If a participant failed an attempt (eg, in the lumbopelvic stability tests, a failed attempt was defined as being able to assume the required initial position but not being able to maintain proper body alignment throughout the entire trial, assuming the initial position but discontinuing the task before completion, or not being able to assume the required initial position), the attempt was classified as invalid and repeated. Only the failed attempt was repeated, not the entire test sequence. If the participant failed again or reported being unable to safely perform the task, the trial was recorded as “not feasible,” and no further attempts were requested for that specific condition. This procedure was applied consistently across all tests.

For the main analyses, we used a peak-performance criterion based on session 2 for each test condition. This approach was selected to emphasize participants’ optimal task execution while limiting the impact of temporary, nonsystematic fluctuations in performance (eg, momentary attentional variation or short-term fatigue), which may occur during physically demanding tasks in older adults. Accordingly, for the lumbopelvic stability tests and for the hip-width and tandem stance posturographic-field tests, the best of the 2 recorded trials (ie, the lowest mean acceleration) was selected for further analyses. For the unstable sitting and the tandem stance dynamic posturographic laboratory tests, the average of the 2 best attempts (ie, lowest mean radial error) was used, based on 5 and 3 trials, respectively. In the straight-line gait tests, the best of the 2 recorded trials (ie, highest speed and percentage values) was used. Finally, for the modified Timed Up and Go test, participants performed 3 trials, and the best trial (ie, lowest completion time) was retained for statistical analyses.

We acknowledge that peak-performance selection can lead to an overestimation of typical functional capacity in older adults. Consequently, to evaluate the robustness and representativeness of the findings, we complemented these primary analyses with sensitivity analyses based on mean performance averaged across sessions 1 and 2. For these sensitivity analyses, performance was computed as the mean of all recorded trials within each session and then averaged across sessions. The only exception was the unstable sitting posturographic test, for which mean performance was calculated as the average of the three best attempts out of 5 trials.

### Statistical Analyses

Statistical analyses were performed to address the study hypotheses and evaluate the reliability of the measurements. Data (presented as mean and SD) from those participants who carried out all the testing sessions were used for the statistical analyses. The normality of the data distribution was explored through the Shapiro-Wilk test. Since the data did not follow a normal distribution, nonparametric statistics were applied. The Wilcoxon test was performed for each test score to explore the existence of statistically significant mean differences between sessions, and the Spearman’s correlation coefficient was used to analyze the relationship between the variables.

In order to avoid possible bias due to the low consistency of certain variables, typical error (TE) and intraclass correlation coefficient (ICC_3,1_) were calculated (confidence limits set at 95%) to evaluate the absolute and relative test-retest reliability, respectively, and to determine which variables could be used in subsequent correlational analyses. The TE was calculated as the SD of the difference between the 2 testing sessions divided by √2. Furthermore, TE values were also expressed as percentages to facilitate data extrapolation, interpretation, and comparison with the pertinent literature. Percentage TE was calculated as the TE×100 and divided by the average of test-retest means. ICC values were interpreted according to the following criteria: poor (<0.50), moderate (0.50‐0.75), good (0.75‐0.90), and excellent (>0.90) [30]. Both absolute and relative reliability indexes were calculated through the spreadsheet proposed by Hopkins [31].

If some variables obtained a poor level of relative reliability [30] (ie, ICC <0.50), the Tukey outlier classification test was applied to the change between sessions. Outliers are based on the IQR (ie, [25th percentile]–1.5×IQR and [75th percentile]+1.5×IQR). The data obtained in the second assessment session for the variables that obtained a moderate-to-excellent level of relative reliability [30] (ie, ICC >0.50) were used to perform a correlation analysis (*r*) between them. Correlational analyses were performed with JASP 0.16.2 software (Eric-Jan Wagenmakers, Department of the Psychological Methods, University of Amsterdam, Nieuwe Achtergracht 129B). The correlation coefficient was interpreted as low (0.30‐0.49), moderate (0.50‐0.69), high (0.70‐0.89), and very high (≥0.90) [32]. In order to minimize the probability of obtaining significant correlations by chance, the prespecified significance level (*P*<.05) was divided by the number of comparisons analyzed (Bonferroni correction).

To examine whether the observed correlations were influenced by the choice of data reduction strategy (session 2 peak-performance vs mean performance averaged across sessions 1 and 2), we conducted complementary sensitivity analyses as described in the previous section. First, we repeated the reliability analyses using mean performance across attempts within each session. Second, we repeated the correlational analyses using the average of session 1 and session 2 mean performance as a more representative estimate of typical performance and to reduce potential measurement noise. The pattern of results across these analyses was then compared with the original session 2 peak-performance analyses.

Given that the lumbopelvic stability tests represent the primary outcome of the study and that they are also the tests most affected by feasibility constraints at higher difficulty levels, an additional focused sensitivity analysis was performed to determine whether test-retest reliability metrics and the key correlations observed in the main analyses based on the session 2 peak-performance criterion were robust enough when analyses were restricted to a more homogeneous group of participants with higher physical fitness who successfully completed all 3 test levels.

## Results

The reliability analyses demonstrated varying levels of test-retest reliability across the assessed variables. Trunk stability tests generally showed a moderate-to-excellent reliability (0.68≤ICC≤0.88; 12.44%≤TE≤31.54%; Table S2 in Multimedia Appendix 1) with the exception of the easiest variations of the back bridge (with double-leg support) and the preferred side bridge (with short double-leg support) and the easiest and most difficult variation of the bird-dog (3-limb position and 2-limb position with the forefoot of the supporting leg elevated, respectively), which showed a poor level of reliability (0.19≤ICC≤0.39, 29.24%≤TE≤38.55%; Table S2 in Multimedia Appendix 1). A moderate-to-excellent level of reliability was shown regarding the reliability analyses for the whole-body balance and gait-related tests (0.51≤ICC≤0.85; 2.55%≤TE≤29.27%; Table S3 in Multimedia Appendix 1) except for the tandem on a firm surface with eyes closed static balance test, the periodicity index of the head-forward gait test, and the head turning gait test (0.37≤ICC≤0.43; 3.06%≤TE≤29.98%; Table S3 in Multimedia Appendix 1). These reliability values, particularly those for the tandem on firm surface and eyes closed static balance tests and the gait test periodicity index, were obtained after the application of the Tukey outlier classification test to address initial low reliability. The Wilcoxon test showed significant between-sessions differences for the mean radial error during the unstable sitting posturographic test and the tandem stance posturographic test, the accelerations of some lumbopelvic stability tests (side bridge with long double-leg support and 2-limb bird-dog positions), the whole-body static balance test with feet hip-width apart on firm surface and eyes open, and the walking speed during all the straight-line gait tests. These showed that there was a learning or repetition effect in these tests and therefore a significantly higher performance in these tests in session 2. For in-depth results, Table S2 in Multimedia Appendix 1 and Table S3 in Multimedia Appendix 1 show the descriptive statistics and the absolute and relative between-session reliability for trunk stability and whole-body balance, gait, and functional mobility tests, respectively.

**Figure 2.**
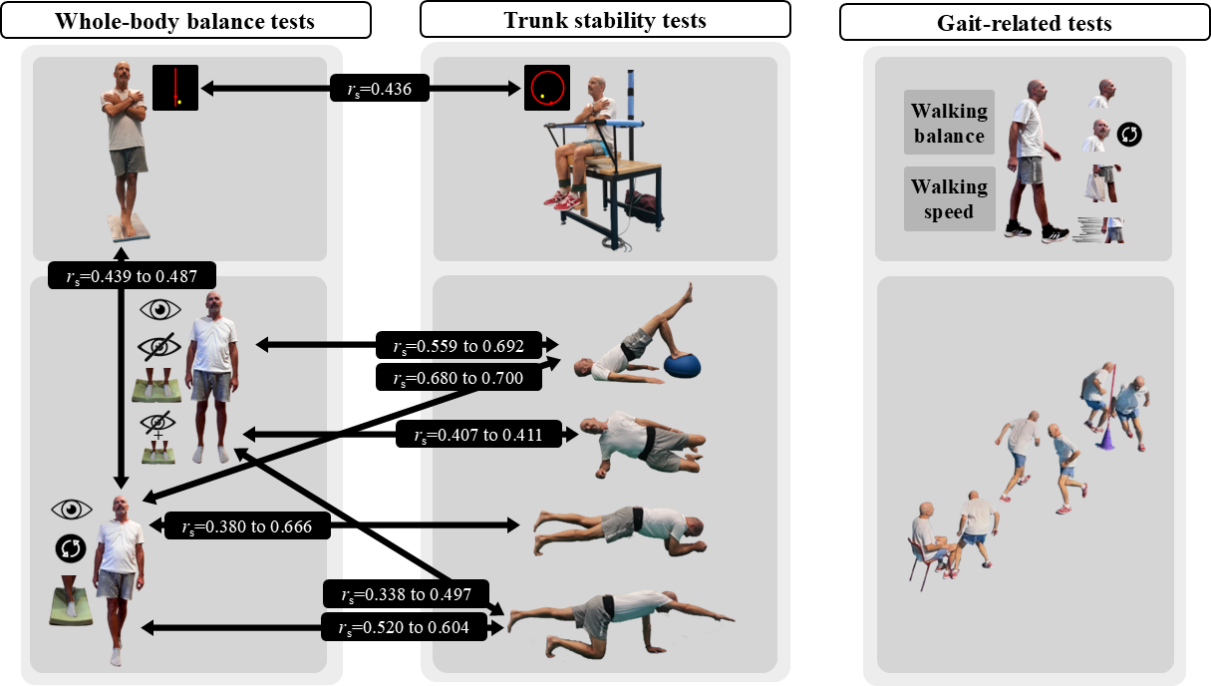
Main correlations between trunk stability tests, whole-body balance tests, and gait-related tests in physically active older adults. The bridge positions displayed represent those that showed the greatest number of correlations with the methodology used to evaluate whole-body static balance in field settings. The 2-limb bird-dog position was included because it was the only one with reliable values. The *tandem stance posturographic test* during a dynamic tracking task showed low but statistically significant correlations with the *tandem stance posturographic-field tests* under steady-state conditions, but no significant correlations with the *hip-width stance posturographic-field tests*. Overall, correlations between the *unstable sitting posturographic test* and the *lumbopelvic stability tests* were not statistically significant. The *unstable sitting* and *tandem stance posturographic tests* during a dynamic tracking task were lowly correlated. *Lumbopelvic stability tests* demonstrated low-to-high correlations with several whole-body balance tests under steady-state conditions, particularly for the back bridge and bird-dog positions. No significant associations were found between trunk stability tests and gait-related tests. Written informed consent was obtained for the publication of the participant’s images.

Given the established reliability and observed learning effects, subsequent correlational analyses were performed using data from the second assessment session. These analyses explored relationships within and between different capabilities, as detailed below. Correlational analyses between the trunk stability tests (Figure 2) generally revealed no statistically significant relationships (*P*<.05) in the moderate-to-high range between the unstable sitting posturographic test and the lumbopelvic stability tests (Table S4 in Multimedia Appendix 1).

As seen in Figure 2, broadly, no statistically significant relationships (*P*<.05) in the moderate-to-high range were found between the tandem stance posturographic test and the whole-body static balance tests. Focusing on some significant comparisons, whole-body static balance tests in the tandem position had a low significant relationship with the tandem stance posturographic test (0.439≤*r*≤0.487; *P*<.05; Table S5 in Multimedia Appendix 1).

Concerning the correlation analysis of the trunk stability tests with the whole-body balance performance tests, two of the four comparisons showed a significant association (Figure 2). A low significant association (*r*=0.436; *P*<.013) was found when comparing the unstable sitting posturographic test with the tandem stance posturographic test. A range of significant associations, from low to high (0.380≤*r*≤0.700; *P*<.013; Table S6 in Multimedia Appendix 1), was observed when lumbopelvic stability tests were compared with whole-body static balance tests (Figure 2). Specifically, moderate correlations (0.559≤ *r*≤0.643; *P*<.013) were found during the back bridge position with the 4 variations of the static balance tests in the hip-width position, some approaching high levels (0.673≤*r*≤0.692; *P*<.013). In addition, the most difficult back bridge (with single-leg support on a hemisphere ball) showed a high correlation with the moderate (firm surface and turning the head) and high (compliant surface and eyes open) difficulty tandem positions (0.680≤*r*≤0.700; *P*<.013). A low correlation was found with the variations in the hip-width position (0.338≤*r*≤0.497; *P*<.013) during the bird-dog position, and there was a moderate correlation with the variations in tandem position (0.520≤*r*≤0.604; *P*<.013). A low correlation was found with the low (firm surface and eyes open) and moderate (firm surface and turning the head) variations in tandem position (0.380≤*r*≤0.427; *P*<.013) during the low (with short double-leg support and arms on hemisphere ball) and moderate (with long double-leg support) variations of front bridge position. The most difficult front bridge (with long single-leg support) showed a high correlation with the most difficult tandem position (compliant surface and eyes open; *r*=0.666; *P*<.013).

Finally, no association was found between trunk stability and any gait-related test (Figure 2). The whole-body static balance tests in the hip-width position showed a consistently low association with walking speed in two of the four straight-line gait tests (0.377≤*r*≤0.407; *P*<.006) and a low-moderate inverse association with the modified Timed Up and Go test (−0.501≤*r*≤–0.444; *P*<.006; Table S7 in Multimedia Appendix 1). This inverse relationship suggests that those participants with higher acceleration values (indicating worse balance control) tended to be faster in these gait-related tests. To try to understand this association better, it seems to be necessary to know the association of height (as a representation of the human inverted pendulum model) with whole-body static balance tests in the hip-width position (0.366≤*r*≤0.489; *P*<.008), with the modified Timed Up and Go test (*r*=−0.527; *P*<.001) and with the walking speed in the 1-sided 10% body weight load test and in the fast gait without running straight-line gait test (0.451≤*r*≤0.503; *P*<.001).

Sensitivity analyses of test-retest reliability based on mean performance are provided in Tables S8-S9 in Multimedia Appendix 1. Overall, reliability estimates were largely comparable to those obtained using the peak-performance approach, supporting the robustness of the measurement properties across different data reduction strategies. Sensitivity analyses of the correlational findings using mean performance and the average across both sessions revealed a consistent overall pattern of results, with the original analysis based on peak performance from session 2. Most associations between trunk stability, whole-body balance, and gait-related outcomes remained low and task-specific. However, a small number of correlations increased from nonsignificant or low to low-moderate and reached statistical significance (Figure 2 vs Figure S1 in Multimedia Appendix 1). Specifically, low but significant associations emerged between the unstable sitting posturographic test and the front bridge with long single-leg support position as well as with the 2-limb bird-dog position of the lumbopelvic stability tests. In addition, the association between the unstable sitting and the tandem stance dynamic posturographic laboratory tests increased from low to moderate, and small but significant associations were observed between these 2 latter tests and the modified Timed Up and Go test (Tables S10-S13 and Figure S1 in Multimedia Appendix 1). These changes are interpreted as the expected consequence of using more stable estimates of performance, which reduce random measurement error and day-to-day variability. Consequently, the primary findings and qualitative interpretation of the results remain unchanged, supporting predominantly low, selective, and task-dependent relationships between trunk stability, balance, and gait-related performance.

The additional focused sensitivity analysis conducted exclusively in participants who were able to successfully complete all 3 test levels yielded results consistent with the main analyses. For each lumbopelvic stability test position, analyses were restricted to the subset of participants who successfully completed all 3 difficulty levels within that test, and test-retest reliability metrics were recomputed accordingly. Although some estimates were attenuated due to a reduced between-subject variability, the overall directional pattern and structure of the findings were preserved. Variations demonstrating good-to-excellent reliability in the full sample retained comparable ICC values in the restricted subsamples, whereas variations with poorer reliability did not systematically improve (Tables S14 in Multimedia Appendix 1). Likewise, associations between trunk stability and whole-body balance were not strengthened (Tables S15 in Multimedia Appendix 1), indicating that the relationships observed in the main analyses were not driven by differences in physical capacity or task feasibility and supporting the robustness of both the reliability estimates and the observed associations.

## Discussion

### Principal Findings

The primary findings of this study highlight a significant association between superior lumbopelvic control during specific bridge and bird-dog exercises and enhanced whole-body static balance. Conversely, no such associations were observed between trunk stability and whole-body dynamic balance or gait-related outcomes. Importantly, consistent with existing literature [5,23], the lack of correlation between trunk stability and whole-body balance in laboratory and field tests underscores a critical point for sport science professionals and practitioners: the direct extrapolation of data obtained with the tools available in field settings to laboratory findings remains unfeasible.

### Influence of Trunk Stability on Whole-Body Static Balance: Task Specificity

Our main results confirmed the first hypothesis, so maintaining a static position in floor-based lumbopelvic stability exercises was associated with the capability to maintain a static upright posture [33]. Thus, incorporating these trunk stability exercises, which require trunk, shoulder, and leg strength to reach the posture, short-term muscle endurance, and muscle coactivation for postural control (via feedforward and/or feedback mechanisms) [34], into a workout routine may enhance whole-body static balance in older adults. However, not all stability exercises showed the same degree of association. Back bridge and bird-dog variations showed more and stronger correlations, emphasizing the role of the trunk-hip extensor (ie, some of the antigravity muscles of the posterior kinetic chain) to maintain static posture. These associations were more evident when balance was increasingly challenged by narrowing the support base through changes in foot position (tandem vs hip-width position), suggesting a greater involvement of the lumbopelvic complex. The asymmetrical tandem stance tends to induce body rotation and lateral adjustments, which may align with the demands of the single-leg back bridge and conventional bird-dog, both involving asymmetry, rotation, and multiplanar control. Conversely, frontal and lateral bridge variations showed fewer and lower correlations. The lack of relevant correlations for the side bridge variations was particularly unexpected as tandem stance tasks seem to have high frontal plane demands and previous studies have linked mediolateral balance to trunk control [34,35]. These results may be due to the fact that control during lateral bridges is heavily mediated by the physical condition of the shoulder complex [36], which could be more affected in older adults by age-related issues such as sarcopenia, tendinopathies, and arthritis [37].

While a significant positive correlation was observed between the well-established trunk stability and whole-body dynamic balance laboratory tests, our second hypothesis was partially rejected, as the strength of these correlations was unexpectedly low (*r*=0.436). This suggests that, although a statistical link exists, trunk stability as measured by the unstable sitting posturographic test may only have a limited practical impact on whole-body dynamic balance in physically active older adults, particularly compared to populations with greater balance deficits in which stronger associations have been reported [26]. In this sense, tandem stance performance showed stronger associations with trunk stability in people with multiple sclerosis [26] (who present neuromuscular deficits) when balance was greatly challenged by placing the weaker leg behind (*r*=0.540; *P*<.05), compared to when the stronger leg was behind (*r*=0.433; *P*<.05), suggesting a greater trunk involvement when postural demands increase. In our sample of physically active older adults, the demands in the unstable sitting posturographic test may not be as determinant for whole-body dynamic balance compared with more disabled populations. In this sense, once a minimum level of trunk stability is reached in a seated position, it no longer contributes to improving whole-body dynamic balance in tandem stance unless a greater balance challenge is imposed on this population [23]. This interpretation should be taken with caution, as higher associations were found (*r*>0.629; *P*<.05) in other samples of older adults of similar characteristics [38], suggesting that the role of trunk stability may have a different impact on whole-body dynamic balance in other scenarios.

Finally, as expected and given the lack of significant associations, maintaining static positions during bridging and bird-dog exercises seems to play a limited role in whole-body dynamic balance during voluntary weight-shifting tasks and also voluntary control during the unstable sitting posturographic test seems less relevant for whole-body static balance. The complex scenario presented in this section aligns with a previous study [39] that confirmed the involvement of task-specific cortical and subcortical pathways after analyzing the excitability of corticospinal inputs to trunk muscles during both voluntary and involuntary tasks. The study [39] also highlighted that this involvement could not be easily predicted based solely on whether the task was classified as voluntary or more “automatic” in nature. This highlights the need for personalized trunk stability assessments that take into account individual characteristics and the underlying neurophysiological mechanisms related to task demands, which may functionally impact whole-body balance in older adults.

### Trunk Stability and Gait-Related Outcomes: Absence of Association

As expected, no association was observed between trunk stability and gait-related outcomes. However, the third hypothesis was partially rejected, as the anticipated correlation between the unstable sitting posturographic test and the modified Timed Up and Go test was not observed. As a previous study on physically active older adults showed [38], trunk stability does not seem to play a significant role in gait performance or even in a gait-related task, such as the Timed Up and Go test, which requires standing, sitting, and turning actions in this population. A previous study in minimally and moderately impaired people with multiple sclerosis [26] found an association with the Timed Up and Go test but not with a straight-line gait test, which is justified by the greater relevance of trunk control needed for its performance. Therefore, in populations with neuromuscular deficits, voluntary trunk control may have a more relevant role during the sit-to-stand, turn, and stand-to-sit phases of the Timed Up and Go test. Again, this interpretation should be taken with caution, as another study involving individuals with greater disability, such as those with hemiparesis following a stroke [40], found no association between an unstable sitting posturographic test and the Timed Up and Go test.

The seemingly counterintuitive inverse association between whole-body static balance (higher acceleration indicating worse control) and walking speed (straight-line gait and Timed Up and Go) might be influenced by anthropometric factors, such as participant height. Taller individuals, while potentially achieving higher walking speeds due to longer strides, often face greater challenges in maintaining static postural control due to a higher center of mass and increased pendulum-like dynamics [41]. This suggests a complex interplay wherein static balance demands differ from dynamic mobility requirements, particularly during phases like sit-to-stand, turning, and stand-to-sit in the Timed Up and Go test [42].

### Discrepancy Between Field and Laboratory Assessments

The lack of correlation between tests grouped under the same capability (ie, trunk stability and whole-body balance) reinforces the notion of the multidimensional nature of postural control in older adults, as previously documented in studies involving other populations [5,23]. Therefore, sport science professionals and practitioners should be aware that the lack of correlations between the novel inertial-sensor–based field tools (CoreMaker and G&B apps) and the well-established force-platform-based assessments makes it unfeasible to extrapolate research results found through laboratory tests to the field. Despite the lack of relationship between laboratory and field tools, the technologically based trunk stability and balance assessment through field tools such as CoreMaker and G&B apps will enhance experimental studies, allowing postural control during training exercises to be assessed in larger, more representative samples, leading to more meaningful clinical findings [21]. In addition, measuring trunk stability during the exercises used for training may allow us to determine to what extent the potential benefits caused by trunk-focused exercise programs (bridges and bird-dogs [24,25]) on balance and gait may be due to an improvement in trunk stability.

### Study Limitations

As with any research, it is important to highlight some limitations. A much larger sample would be desirable in the most difficult variations of bridges (Table S2 in Multimedia Appendix 1) to minimize the probability of a type II error due to undersampling. Second, our results are based on a sample of physically active older adults and of both sexes, so future studies would need to examine the relationships of these tests in more fragile populations and also potential sex differences. Although our study focused on well-established force platform and inertial sensor protocols to assess trunk stability, balance, and gait, it is important to admit that other assessment tools exist and that using different tests might have led to different results. Future studies should analyze and develop field tests to evaluate the reactive control of the trunk due to its apparent potential relationship with the mechanisms required during a fall situation in older people. Moreover, trunk-focused stability outcomes should also be analyzed during specific tests designed to quantify whole-body balance, gait, and functional mobility, particularly under conditions imposing high demands on trunk control, to better understand how performance in these tasks relates to direct measurements of trunk involvement [42,43]. Although for the accelerometry-based field tests postural control was summarized using the average of the acceleration magnitude data series obtained from the 3-axis as a proxy for performance, future studies with more focused designs should incorporate axis-specific and advanced signal analyses to further elucidate control mechanisms during the studied tasks. Although the main conclusions were robust across multiple analytical strategies and the qualitative interpretation of the results remained similar, as with any complex analytical framework, the choice of data reduction strategy may have had a modest influence on the magnitude of some associations. Importantly, the convergence of findings across these contrasting analytical strategies strengthens confidence in the robustness, task-specificity, and interpretability of the observed relationships, indicating that the primary analysis represents a conservative estimate rather than a biased one.

### Conclusions

This study provides novel evidence linking lumbopelvic control in floor-based exercises (mainly back bridge and bird-dog) to whole-body static balance in physically active older adults, supporting trunk stability as a crucial factor for maintaining steady standing positions in physically active older adults. Crucially, this research highlights a notable disconnection between novel inertial sensor–based field assessments (CoreMaker and G&B apps) and traditional force platform–based laboratory protocols for trunk stability and balance. While direct extrapolation between these methods is not advisable, the accessibility of field tools like CoreMaker offers a promising avenue [21] for standardized lumbopelvic stability assessment and the monitoring of training load in broader populations. Future interventions targeting static balance in older adults should consider incorporating specific bridging and bird-dog positions [24,25] to enhance trunk control.

## Supplementary material

10.2196/83546 Multimedia Appendix 1 Clinical data, reliability, associations, and sensitivity analyses.

10.2196/83546 Checklist 1 STROBE checklist.

## Abbreviations

ICC: intraclass correlation coefficient
STROBE: Strengthening the Reporting of Observational Studies in Epidemiology
TE: typical error

## References

[1] Seidler RD, Bernard JA, Burutolu TB (2010). Motor control and aging: links to age-related brain structural, functional, and biochemical effects. Neurosci Biobehav Rev.

[2] Haddas R, Satin A, Lieberman I (2020). What is actually happening inside the “cone of economy”: compensatory mechanisms during a dynamic balance test. Eur Spine J.

[3] Quirk DA, Hubley-Kozey CL (2014). Age-related changes in trunk neuromuscular activation patterns during a controlled functional transfer task include amplitude and temporal synergies. Hum Mov Sci.

[4] McGill A, Houston S, Lee RYW, Gao Y (2019). Effects of a ballet intervention on trunk coordination and range of motion during gait in people with Parkinson’s. Cogent Med.

[5] Vera-Garcia FJ, López-Plaza D, Juan-Recio C, Barbado D (2019). Tests to measure core stability in laboratory and field settings: reliability and correlation analyses. J Appl Biomech.

[6] Saito K, Matsunaga T, Iwami T, Shimada Y (2014). Evaluation of trunk stability in the sitting position using a new device. Biomed Res.

[7] Akizuki K, Echizenya Y, Kaneno T, Ohashi Y (2019). Usefulness of an unstable board balance test to accurately identify community-dwelling elderly individuals with a history of falls. J Rehabil Med.

[8] Monteiro PHM, Valenciano PJ, Mendes PHS, Teixeira LA (2025). Association of 30-s sit-to-stand power test outcome with body balance in physically active older adults. J Aging Phys Act.

[9] Valenciano PJ, Castan VE, Monteiro PHM, Teixeira LA (2024). Symmetric unipedal balance in quiet stance and dynamic tasks in older individuals. Brain Res.

[10] Valenciano PJ, Monteiro PHM, Lazzaro IM (2024). Validation of the Equidyn protocol for evaluation of dynamic balance in older adults through a smartphone application. Gait Posture.

[11] Granacher U, Gollhofer A, Hortobágyi T, Kressig RW, Muehlbauer T (2013). The importance of trunk muscle strength for balance, functional performance, and fall prevention in seniors: a systematic review. Sports Med.

[12] Granacher U, Lacroix A, Roettger K, Gollhofer A, Muehlbauer T (2014). Relationships between trunk muscle strength, spinal mobility, and balance performance in older adults. J Aging Phys Act.

[13] Suri P, Kiely DK, Leveille SG, Frontera WR, Bean JF (2009). Trunk muscle attributes are associated with balance and mobility in older adults: a pilot study. PM R.

[14] Stotz A, Mason J, Groll A, Zech A (2023). Which trunk muscle parameter is the best predictor for physical function in older adults?. Heliyon.

[15] Acar E, Çankaya T, Öner S (2020). The relationship between trunk muscle thickness and static postural balance in older adults. J Aging Phys Act.

[16] Shahtahmassebi B, Hebert JJ, Hecimovich MD, Fairchild TJ (2017). Associations between trunk muscle morphology, strength and function in older adults. Sci Rep.

[17] Zhai M, Huang Y, Zhou S (2023). Effects of age-related changes in trunk and lower limb range of motion on gait. BMC Musculoskelet Disord.

[18] Haddas R, Hu X, Lieberman IH (2020). The correlation of spinopelvic parameters with biomechanical parameters measured by gait and balance analyses in patients with adult degenerative scoliosis. Clin Spine Surg.

[19] Takahashi Y, Saito K, Matsunaga T (2020). Relationship between dynamic trunk balance and the Balance Evaluation Systems Test in elderly women. Prog Rehabil Med.

[20] Lee KYT, Hui-Chan CWY, Tsang WWN (2016). Reliability and validity of the sequential weight-shifting test: a new functional approach to the assessment of the sitting balance of older adults. J Phys Ther Sci.

[21] Bohlke K, Redfern MS, Rosso AL, Sejdic E (2023). Accelerometry applications and methods to assess standing balance in older adults and mobility-limited patient populations: a narrative review. Aging Clin Exp Res.

[22] Heredia-Elvar JR, Juan-Recio C, Prat-Luri A, Barbado D, de los Ríos-Calonge J, Vera-Garcia FJ (2024). Exercise intensity progressions and criteria to prescribe core stability exercises in young physically active men: a smartphone accelerometer-based study. J Strength Cond Res.

[23] De Los Ríos‐Calonge J, Barbado D, Prat‐Luri A (2024). Are trunk stability and endurance determinant factors for whole‐body dynamic balance in physically active young males? A multidimensional analysis. Scandinavian J Med Sci Sports.

[24] Granacher U, Lacroix A, Muehlbauer T, Roettger K, Gollhofer A (2013). Effects of core instability strength training on trunk muscle strength, spinal mobility, dynamic balance and functional mobility in older adults. Gerontology.

[25] Lima M, Silva B, Rocha-Rodrigues S, Bezerra P (2021). The impact of an 8-week pilates-based physical training program on functional mobility: data from a septuagenarian group. Biomed Hum Kinet.

[26] Barbado D, Gomez-Illan R, Moreno-Navarro P, Valero-Conesa G, Reina R, Vera-Garcia FJ (2020). Postural control quantification in minimally and moderately impaired persons with multiple sclerosis: the reliability of a posturographic test and its relationships with functional ability. J Sport Health Sci.

[27] Rashid U, Barbado D, Olsen S (2021). Validity and reliability of a smartphone app for gait and balance assessment. Sensors (Basel).

[28] Christopher A, Kraft E, Olenick H, Kiesling R, Doty A (2021). The reliability and validity of the Timed Up and Go as a clinical tool in individuals with and without disabilities across a lifespan: a systematic review. Disabil Rehabil.

[29] Winter DA (2009). Biomechanics and Motor Control of Human Movement.

[30] Koo TK, Li MY (2016). A guideline of selecting and reporting intraclass correlation coefficients for reliability research. J Chiropr Med.

[31] Hopkins WG (2015). Spreadsheets for Analysis of Validity and Reliability. Sportscience.

[32] Hinkle DE, Wiersma W, Jurs SG (2003). Applied Statistics for the Behavioral Sciences.

[33] Kiss R, Schedler S, Muehlbauer T (2018). Associations between types of balance performance in healthy individuals across the lifespan: a systematic review and meta-analysis. Front Physiol.

[34] Shumway-Cook A, Woollacott MH (2007). Motor Control: Translating Research Into Clinical Practice.

[35] Haruyama K, Kasai K, Makino R, Hoshi F, Nishihara K (2019). Quantification of trunk segmental coordination and head stability in laterally unstable sitting identifies aging and cerebellar ataxia. Clin Biomech.

[36] Juan-Recio C, Prat-Luri A, Galindo A, Manresa-Rocamora A, Barbado D, Vera-Garcia FJ (2022). Is the side bridge test valid and reliable for assessing trunk lateral flexor endurance in recreational female athletes?. Biology (Basel).

[37] Minetto MA, Giannini A, McConnell R, Busso C, Torre G, Massazza G (2020). Common musculoskeletal disorders in the elderly: the Star triad. J Clin Med.

[38] Hernández-Sánchez S, De Los Ríos-Calonge J, Juan-Recio C, Prat-Luri A, Vera-Garcia FJ, Barbado D (2026). Understanding the effects of aging on whole-body dynamic balance, trunk stability, functional mobility, and hip strength. Sci Rep.

[39] Chiou SY, Gottardi SEA, Hodges PW, Strutton PH (2016). Corticospinal excitability of trunk muscles during different postural tasks. PLoS ONE.

[40] Bruyneel AV, Mesure S, Reinmann A (2022). Validity and reliability of center of pressure measures to quantify trunk control ability in individuals after stroke in subacute phase during unstable sitting test. Heliyon.

[41] Alonso AC, Mochizuki L, Silva Luna NM, Ayama S, Canonica AC, Greve JMDA (2015). Relation between the sensory and anthropometric variables in the quiet standing postural control: is the inverted pendulum important for the static balance control?. Biomed Res Int.

[42] Rezende LS, Monteiro PH, Oliveira JA (2023). Do Timed Up and Go and five times sit to stand test outcomes correlate with trunk stability? A pilot-study. Braz J Motor Behav.

[43] Naito D, Honda K, Sekiguchi Y, Izumi SI, Ebihara S (2025). Characteristics of trunk acceleration and angular velocity in turning movement in post-stroke patients with high risk of falling. Sensors (Basel).

