# Supplementary material for: Smartphone-Based Field Assessment of Trunk Stability and Its Relationship With Whole-Body Balance in Older Adults: Cross-Sectional Study
Source: JMIR Aging. 2026 May 6;9:e83546. doi: 10.2196/83546 (PMC13148756; doi:10.2196/83546)
Supplement: Multimedia Appendix 1 [file aging-v9-e83546-s001.docx]

| **Table S1. Main clinical data of older adults** | | |  |  |  |  |  |  |  |  |  |
| --- | --- | --- | --- | --- | --- | --- | --- | --- | --- | --- | --- |
|  | **Male** | | |  | **Female** | | |  | **Total** | | |
|  | **n=21** | | |  | **n= 32** | | |  | **n= 53** | | |
|  | N |  | % |  | N |  | % |  | N |  | % |
| Smokers | 1 |  | 5 |  | 1 |  | 3 |  | 2 |  | 4 |
| Falls in the previous 12 months | 2 |  | 10 |  | 5 |  | 16 |  | 7 |  | 13 |
| Diagnosed diseases |  |  |  |  |  |  |  |  |  |  |  |
| *Hypertension* | 10 |  | 48 |  | 10 |  | 31 |  | 20 |  | 38 |
| *Hypercholesterolemia* | 6 |  | 29 |  | 10 |  | 31 |  | 16 |  | 30 |
| *Coronary disease* | 3 |  | 14 |  | 2 |  | 6 |  | 5 |  | 9 |
| *Valvular heart disease/cardiomyopathy* | 0 |  | 0 |  | 1 |  | 3 |  | 1 |  | 2 |
| *Stroke* | 1 |  | 5 |  | 1 |  | 3 |  | 2 |  | 4 |
| *Diabetes* | 2 |  | 10 |  | 1 |  | 3 |  | 3 |  | 6 |
| *Osteoporosis* | 0 |  | 0 |  | 5 |  | 16 |  | 5 |  | 9 |
| *Bone fractures* | 2 |  | 10 |  | 3 |  | 9 |  | 5 |  | 9 |
| *Osteoarthritis or arthritis* | 4 |  | 19 |  | 13 |  | 41 |  | 17 |  | 32 |
| *Cancer* | 0 |  | 0 |  | 2 |  | 6 |  | 2 |  | 4 |
| *Depression* | 2 |  | 10 |  | 0 |  | 0 |  | 2 |  | 4 |
| *Other ^A^* | 5 |  | 24 |  | 9 |  | 28 |  | 14 |  | 26 |
| *None* | 8 |  | 38 |  | 7 |  | 22 |  | 15 |  | 28 |
| Drug intake (%) |  | | |  |  | | |  |  | | |
| *Cardiovascular system* | 10 |  | 48 |  | 14 |  | 44 |  | 24 |  | 45 |
| *Metabolic system* | 9 |  | 43 |  | 16 |  | 50 |  | 25 |  | 47 |
| *Musculoskeletal system* | 1 |  | 5 |  | 8 |  | 25 |  | 9 |  | 17 |
| *Respiratory system* | 0 |  | 0 |  | 1 |  | 3 |  | 1 |  | 2 |
| *Sleeping aids* | 2 |  | 10 |  | 1 |  | 3 |  | 3 |  | 6 |
| *Painkillers and/or antidepressants* | 1 |  | 5 |  | 2 |  | 6 |  | 3 |  | 6 |
| *Other ^B^* | 6 |  | 29 |  | 3 |  | 9 |  | 9 |  | 17 |
| *None* | 6 |  | 29 |  | 5 |  | 16 |  | 11 |  | 21 |
| Discomfort in the previous 6 months |  | | |  |  | | |  |  | | |
| *Upper limb pain* | 3 |  | 14 |  | 6 |  | 19 |  | 9 |  | 17 |
| *Lower limb pain* | 3 |  | 14 |  | 6 |  | 19 |  | 9 |  | 17 |
| *Cervical pain* | 1 |  | 5 |  | 3 |  | 9 |  | 4 |  | 8 |
| *Upper back pain* | 1 |  | 5 |  | 2 |  | 6 |  | 3 |  | 6 |
| *Low back pain* | 10 |  | 48 |  | 13 |  | 41 |  | 23 |  | 43 |
| *Breathing* | 1 |  | 5 |  | 2 |  | 6 |  | 3 |  | 6 |
| *Swollen legs* | 0 |  | 0 |  | 1 |  | 3 |  | 1 |  | 2 |
| *Daily living memory lapses* | 1 |  | 5 |  | 2 |  | 6 |  | 3 |  | 6 |
| *Difficulty focusing* | 1 |  | 5 |  | 2 |  | 6 |  | 3 |  | 6 |
| *Difficulty making decisions* | 1 |  | 5 |  | 1 |  | 3 |  | 2 |  | 4 |
| *Dizziness or vertigo* | 3 |  | 14 |  | 6 |  | 19 |  | 9 |  | 17 |
| *Skin* | 3 |  | 14 |  | 2 |  | 6 |  | 5 |  | 9 |
| *Stomach/intestine* | 1 |  | 5 |  | 3 |  | 9 |  | 4 |  | 8 |
| *Urinary incontinence* | 3 |  | 14 |  | 3 |  | 9 |  | 6 |  | 11 |
| *Other ^c^* | 0 |  | 0 |  | 2 |  | 6 |  | 2 |  | 4 |
| *None* | 7 |  | 33 |  | 7 |  | 22 |  | 14 |  | 26 |
| Injury and/or surgery (%) |  | | |  |  | | |  |  | | |
| *Shoulders* | 1 |  | 5 |  | 5 |  | 16 |  | 6 |  | 11 |
| *Elbows* | 0 |  | 0 |  | 2 |  | 6 |  | 2 |  | 4 |
| *Wrists* | 1 |  | 5 |  | 2 |  | 6 |  | 3 |  | 6 |
| *Back* | 2 |  | 10 |  | 6 |  | 19 |  | 8 |  | 15 |
| *Hips* | 1 |  | 5 |  | 1 |  | 3 |  | 2 |  | 4 |
| *Knees* | 0 |  | 0 |  | 6 |  | 19 |  | 6 |  | 11 |
| *Ankles* | 1 |  | 5 |  | 2 |  | 6 |  | 3 |  | 6 |
| *None* | 16 |  | 76 |  | 18 |  | 56 |  | 34 |  | 64 |
| Vision issues (%) | 8 |  | 38 |  | 17 |  | 53 |  | 25 |  | 47 |
| Hearing issues (%) | 1 |  | 5 |  | 0 |  | 0 |  | 1 |  | 2 |
| *^A^*= Other diagnosed diseases: tachycardia (Pradaxa); psoriasis; hyperuricemia; gastroesophageal reflux disease; ocular hypertension (glaucoma); herpes zoster; systemic sclerosis; benign prostatic hyperplasia; hypothyroidism; systemic lupus erythematosus; thyroid disorder; post-COVID sequelae; ulcerative colitis. *^B^*= Other drug intake: eye drops; prostate medication; medication to aid swallowing; gastric protector; thyroid medication; uric acid medication; ulcerative colitis treatment. *^C^*= Other discomfort: presbyopia; hiatal hernia. | | | | | | | | | | | |
|  | | | | | | | | | | | |

| **Table S2.** Descriptive statistics and absolute and relative between-session reliability for the trunk stability tests. | | | | | | | | | | | | | | | | | | | |
| --- | --- | --- | --- | --- | --- | --- | --- | --- | --- | --- | --- | --- | --- | --- | --- | --- | --- | --- | --- |
|  | |  | n | **Session 1 (mean ± SD)** | | | **Session 2 (mean ± SD)** | | | **Typical error** | | | | | **ICC Mean (LCL - UCL)** | | | |  |
|  |  |  |  |  |  |  |  |  |  | **Mean (LCL - UCL)** | | | | **%** |  |  |  |  |  |
| **Unstable sitting** **posturographic test** (mm) | |  | 53 | 19.60 | ± | 6.42 | 16.18 | ± | 6.25† | 3.43 | 2.88 | - | 4.24 | 19.16 | 0.71 | 0.55 | - | 0.82 |  |
| **Lumbopelvic stability tests** (m/s^2^) | Back bridge with double-leg support | BB_DL_ | 51 | 0.15 | ± | 0.06 | 0.14 | ± | 0.06 | 0.06 | 0.05 | - | 0.07 | 38.55 | 0.19 | -0.09 | - | 0.44 |  |
|  | Back bridge with single-leg support | BB_SL_ | 43 | 0.23 | ± | 0.11 | 0.22 | ± | 0.08 | 0.04 | 0.04 | - | 0.06 | 19.76 | 0.81 | 0.67 | - | 0.89 |  |
|  | Back bridge with single-leg support on a hemisphere ball | BB_SLB_ | 29 | 0.25 | ± | 0.09 | 0.24 | ± | 0.09 | 0.04 | 0.03 | - | 0.05 | 16.20 | 0.83 | 0.66 | - | 0.91 |  |
|  | Side bridge with short double-leg support | SB_SDL_ | 46 | 0.21 | ± | 0.07 | 0.19 | ± | 0.08 | 0.06 | 0.05 | - | 0.07 | 29.29 | 0.39 | 0.12 | - | 0.61 |  |
|  | Side bridge with short single-leg support | SB_SSL_ | 39 | 0.20 | ± | 0.08 | 0.19 | ± | 0.07 | 0.03 | 0.02 | - | 0.03 | 13.62 | 0.87 | 0.77 | - | 0.93 |  |
|  | Side bridge with long double-leg support | SB_LDL_ | 20 | 0.32 | ± | 0.12 | 0.29 | ± | 0.09† | 0.04 | 0.03 | - | 0.06 | 13.13 | 0.87 | 0.71 | - | 0.95 |  |
|  | Front bridge with short double-leg support and arms on a hemisphere ball | FB_SDLB_ | 52 | 0.09 | ± | 0.04 | 0.10 | ± | 0.06 | 0.03 | 0.03 | - | 0.04 | 31.54 | 0.68 | 0.50 | - | 0.80 |  |
|  | Front bridge with long double-leg support | FB_LDL_ | 51 | 0.21 | ± | 0.09 | 0.21 | ± | 0.08 | 0.03 | 0.02 | - | 0.04 | 13.94 | 0.88 | 0.80 | - | 0.93 |  |
|  | Front bridge with long single-leg support | FB_LSL_ | 34 | 0.33 | ± | 0.11 | 0.32 | ± | 0.10 | 0.04 | 0.03 | - | 0.05 | 12.44 | 0.85 | 0.72 | - | 0.92 |  |
|  | Three-limb bird-dog position | BD_3L_ | 53 | 0.13 | ± | 0.05 | 0.12 | ± | 0.04 | 0.04 | 0.03 | - | 0.05 | 29.24 | 0.32 | 0.06 | - | 0.54 |  |
|  | Two-limb bird-dog position | BD_2L_ | 50 | 0.20 | ± | 0.06 | 0.19 | ± | 0.06† | 0.03 | 0.02 | - | 0.03 | 13.29 | 0.83 | 0.72 | - | 0.90 |  |
|  | Two-limb bird-dog position with the forefoot of the supporting leg elevated | BD_2LF_ | 15 | 0.58 | ± | 0.20 | 0.55 | ± | 0.23 | 0.19 | 0.14 | - | 0.29 | 33.03 | 0.27 | -0.26 | - | 0.68 |  |
| SD: standard deviation; ICC: intraclass correlation coefficient; LCL: lower confidence limit set at 95%; UCL: upper confidence limit set at 95%; †Pairwise comparisons between session 1 and 2: significant differences (*P*<.05). | | | | | | | | | | | | | | | | | | | |

| **Table S3.** Descriptive statistics and absolute and relative between-session reliability for the whole-body balance, gait and functional mobility tests. | | | | | | | | | | | | | | | | | | | |  |
| --- | --- | --- | --- | --- | --- | --- | --- | --- | --- | --- | --- | --- | --- | --- | --- | --- | --- | --- | --- | --- |
|  | | |  | n | **Session 1 (mean ± SD)** | | | **Session 2 (mean ± SD)** | | | **Typical error** | | | | | **ICC Mean (LCL - UCL)** | | | | |
|  |  |  |  |  |  |  |  |  |  |  | **Mean (LCL - UCL)** | | | | **%** |  |  |  |  |  |
| **Whole-body dynamic balance test** | **Tandem stance posturographic test** (mm) | |  | 53 | 15.00 | ± | 6.42 | 13.20 | ± | 4.41† | 3.87 | 3.25 | - | 4.79 | 27.47 | 0.51 | 0.28 | - | 0.69 | |
| **Whole-body static balance tests** | **Tests with feet positioned hip-width apart** (m/s^2^) | Hip-width, firm surface and eyes open | H_FSEO_ | 51 | 0.031 | ± | 0.011 | 0.030 | ± | 0.009† | 0.004 | 0.003 | - | 0.005 | 12.96 | 0.85 | 0.75 | - | 0.91 | |
|  |  | Hip-width, firm surface and eyes closed | H_FSEC_ | 51 | 0.034 | ± | 0.013 | 0.033 | ± | 0.014 | 0.006 | 0.005 | - | 0.007 | 16.83 | 0.82 | 0.71 | - | 0.90 | |
|  |  | Hip-width, compliant surface and eyes open | H_CSEO_ | 51 | 0.041 | ± | 0.015 | 0.038 | ± | 0.013 | 0.007 | 0.006 | - | 0.009 | 18.66 | 0.73 | 0.57 | - | 0.84 | |
|  |  | Hip-width, compliant surface and eyes closed | H_CSEC_ | 51 | 0.049 | ± | 0.023 | 0.046 | ± | 0.019 | 0.012 | 0.010 | - | 0.015 | 25.42 | 0.67 | 0.49 | - | 0.80 | |
|  | **Tests with feet positioned in tandem** (m/s^2^) | Tandem, firm surface and eyes open | T_FSEO_ | 47 | 0.11 | ± | 0.04 | 0.11 | ± | 0.06 | 0.03 | 0.03 | - | 0.04 | 28.67 | 0.60 | 0.38 | - | 0.75 | |
|  |  | Tandem, firm surface and turning the head | T_FSTH_ | 45 | 0.15 | ± | 0.06 | 0.17 | ± | 0.08 | 0.03 | 0.03 | - | 0.04 | 21.70 | 0.75 | 0.59 | - | 0.86 | |
|  |  | Tandem, compliant surface and eyes open | T_CSEO_ | 43 | 0.16 | ± | 0.08 | 0.16 | ± | 0.06 | 0.05 | 0.04 | - | 0.06 | 29.27 | 0.51 | 0.25 | - | 0.70 | |
|  |  | Tandem, firm surface and eyes closed | T_FSEC_ | 27 | 0.17 | ± | 0.07 | 0.18 | ± | 0.07 | 0.05 | 0.04 | - | 0.07 | 29.98 | 0.43 | 0.07 | - | 0.69 | |
| **Straight-line gait tests** | **Periodicity index** (%) | Head-forward gait periodicity index | G_HFPI_ | 45 | 69.69 | ± | 2.59 | 70.00 | ± | 2.76 | 2.14 | 1.77 | - | 2.70 | 3.06 | 0.37 | 0.09 | - | 0.60 | |
|  |  | Head turning gait periodicity index | G_HTPI_ | 48 | 66.76 | ± | 4.03 | 67.53 | ± | 4.00 | 3.19 | 2.65 | - | 3.99 | 4.74 | 0.38 | 0.11 | - | 0.60 | |
|  |  | One-sided 10% body weight load gait periodicity index | G_LPI_ | 44 | 68.74 | ± | 2.92 | 68.73 | ± | 2.84 | 1.75 | 1.45 | - | 2.22 | 2.55 | 0.64 | 0.42 | - | 0.78 | |
|  |  | Fast gait without running periodicity index | G_FPI_ | 45 | 69.92 | ± | 3.24 | 68.72 | ± | 4.60 | 2.76 | 2.29 | - | 3.49 | 3.99 | 0.53 | 0.28 | - | 0.71 | |
|  | **Walking speed** (m/s) | Head-forward gait walking speed | G_HFWS_ | 50 | 1.31 | ± | 0.20 | 1.39 | ± | 0.20† | 0.12 | 0.10 | - | 0.16 | 9.26 | 0.62 | 0.42 | - | 0.77 | |
|  |  | Head turning gait walking speed | G_HTWS_ | 50 | 1.16 | ± | 0.25 | 1.28 | ± | 0.22† | 0.15 | 0.13 | - | 0.19 | 12.52 | 0.59 | 0.38 | - | 0.75 | |
|  |  | One-sided 10% body weight load gait walking speed | G_LWS_ | 50 | 1.38 | ± | 0.20 | 1.45 | ± | 0.23† | 0.09 | 0.07 | - | 0.11 | 6.32 | 0.83 | 0.72 | - | 0.90 | |
|  |  | Fast gait without running walking speed | G_FWS_ | 50 | 1.68 | ± | 0.26 | 1.72 | ± | 0.28† | 0.14 | 0.12 | - | 0.18 | 8.25 | 0.74 | 0.58 | - | 0.84 | |
| **Functional mobility test** | **Modified Timed Up & Go test** (s) | |  | 53 | 5.63 | ± | 1.03 | 5.37 | ± | 0.89 | 0.44 | 0.37 | - | 0.54 | 7.98 | 0.80 | 0.67 | - | 0.88 | |
| SD: standard deviation; ICC: intraclass correlation coefficient; LCL: lower confidence limit set at 95%; UCL: upper confidence limit set at 95%; †Pairwise comparisons between session 1 and 2: significant differences (*P*<.05). | | | | | | | | | | | | | | | | | | | |  |

| **Table S4.** Relationship between the two methodologies to evaluate trunk stability. | | | | | | | | | |
| --- | --- | --- | --- | --- | --- | --- | --- | --- | --- |
|  |  | BB_SL_ | BB_SLB_ | SB_SSL_ | SB_LDL_ | FB_SDLB_ | FB_LDL_ | FB_LSL_ | BD_2L_ |
| **Unstable sitting posturographic test** (mm) |  |  |  |  |  |  |  |  |  |
|  |  | -0.121 | -0.044 | 0.005 | 0.179 | 0.289* | -0.010 | 0.129 | 0.275 |
| **Lumbopelvic stability tests** (m/s^2^) |  |  |  |  |  |  |  |  |  |
| Back bridge with single-leg support | BB_SL_ | - | 0.694* | 0.469* | 0.311 | 0.379* | 0.205 | 0.367* | 0.597* |
| Back bridge with single-leg support on a hemisphere ball | BB_SLB_ |  | - | 0.520* | 0.218 | 0.688* | 0.251 | 0.619* | 0.595* |
| Side bridge with short single-leg support | SB_SSL_ |  |  | - | 0.721* | 0.564* | 0.532* | 0.656* | 0.424* |
| Side bridge with long double-leg support | SB_LDL_ |  |  |  | - | 0.583* | 0.701* | 0.551* | 0.474* |
| Front bridge with short double-leg support and arms on hemisphere ball | FB_SDLB_ |  |  |  |  | - | 0.433* | 0.594* | 0.491* |
| Front bridge with long double-leg support | FB_LDL_ |  |  |  |  |  | - | 0.620* | 0.377* |
| Front bridge with long single-leg support | FB_LSL_ |  |  |  |  |  |  | - | 0.513* |
| Two-limb bird-dog position | BD_2L_ |  |  |  |  |  |  |  | - |
| One comparison; statistical significance level for correlation: * *P*<.05. | | | | | | | | | |

| **Table S5.** Relationship between the two methodologies to evaluate whole-body balance. | | | | | | | | | | | |  |
| --- | --- | --- | --- | --- | --- | --- | --- | --- | --- | --- | --- | --- |
|  | | | | | **Whole-body static balance tests** | | | | | | | |
|  |  |  |  |  | **Tests with feet positioned**  **hip-width apart** (m/s^2^) | | | | **Tests with feet positioned in tandem** (m/s^2^) | | | |
|  |  | | |  | H_FSEO_ | H_FSEC_ | H_CSEO_ | H_CSEC_ | T_FSEO_ | T_FSTH_ | T_CSEO_ | |
| **Whole-body dynamic balance test** | **Tandem stance posturographic test** (mm) | | |  |  |  |  |  |  |  |  | |
|  |  |  |  |  | 0.114 | 0.097 | 0.110 | 0.036 | 0.462* | 0.487* | 0.439* | |
| **Whole-body static balance tests** | **Tests** **with feet positioned hip-width apart** (m/s^2^) | | | |  |  |  |  |  |  |  | |
|  | Hip-width, firm surface and eyes open | | | H_FSEO_ | - | 0.943* | 0.852* | 0.598* | 0.563* | 0.573* | 0.439* | |
|  | Hip-width, firm surface and eyes closed | | | H_FSEC_ |  | - | 0.849* | 0.673* | 0.505* | 0.502* | 0.364* | |
|  | Hip-width, compliant surface and eyes open | | | H_CSEO_ |  |  | - | 0.771* | 0.605* | 0.612* | 0.545* | |
|  | Hip-width, compliant surface and eyes closed | | | H_CSEC_ |  |  |  | - | 0.444* | 0.434* | 0.455* | |
|  | **Tests** **with** **feet positioned in tandem** (m/s^2^) | | |  |  |  |  |  |  |  |  | |
|  | Tandem, firm surface and eyes open | | | T_FSEO_ |  |  |  |  | - | 0.860* | 0.676* | |
|  | Tandem, firm surface and turning the head | | | T_FSTH_ |  |  |  |  |  | - | 0.703* | |
|  | Tandem, compliant surface and eyes open | | | T_CSEO_ |  |  |  |  |  |  | - | |
| One comparison; statistical significance level for correlation: * *P*<.05. | | | | | | | | | | | | |

| **Table S6.** Relationship between the two assessment methods representing trunk stability and the two representing whole-body balance. | | | | | | | | | |
| --- | --- | --- | --- | --- | --- | --- | --- | --- | --- |
|  |  | **Whole-body dynamic balance test** | **Whole-body static balance tests** | | | | | | |
|  |  |  | **Tests with feet positioned hip-width apart** (m/s^2^) | | | | **Tests with feet positioned in tandem** (m/s^2^) | | |
|  |  | **Tandem stance posturographic test** (mm) | H_FSEO_ | H_FSEC_ | H_CSEO_ | H_CSEC_ | T_FSEO_ | T_FSTH_ | T_CSEO_ |
| **Unstable sitting posturographic test** (mm) | |  |  |  |  |  |  |  |  |
|  |  | 0.436* | -0.027 | -0.038 | -0.066 | -0.109 | 0.263 | 0.161 | 0.212 |
| **Lumbopelvic stability tests** (m/s^2^) | |  |  |  |  |  |  |  |  |
|  | BB_SL_ | 0.021 | 0.601* | 0.588* | 0.559* | 0.468* | 0.432* | 0.342 | 0.278 |
|  | BB_SLB_ | 0.066 | 0.643* | 0.579* | 0.692* | 0.673* | 0.458 | 0.680* | 0.700* |
|  | SB_SSL_ | 0.035 | 0.407* | 0.411* | 0.281 | 0.307 | 0.348 | 0.218 | 0.302 |
|  | SB_LDL_ | -0.162 | 0.148 | 0.044 | -0.073 | 0.011 | 0.404 | 0.132 | 0.111 |
|  | FB_SDLB_ | 0.010 | 0.272 | 0.261 | 0.306 | 0.156 | 0.380* | 0.413* | 0.165 |
|  | FB_LDL_ | 0.178 | 0.119 | 0.140 | 0.215 | 0.227 | 0.427* | 0.389* | 0.236 |
|  | FB_LSL_ | 0.149 | 0.416 | 0.337 | 0.437 | 0.497* | 0.380 | 0.425 | 0.666* |
|  | BD_2L_ | 0.350 | 0.388* | 0.347 | 0.497* | 0.444* | 0.604* | 0.555* | 0.520* |
| The lumbopelvic stability test positions: back bridge with single-leg support (BB_SL_); back bridge with single-leg support on a hemisphere ball (BB_SLB_); side bridge with short single-leg support (SB_SSL_); side bridge with long double-leg support (SB_LDL_); front bridge with short double-leg support and arms on hemisphere ball (FB_SDLB_); front bridge with long double-leg support (FB_LDL_); front bridge with long single-leg support (FB_LSL_); two-limb bird-dog position (BD_2L_). Whole-body static balance tests: hip-width, firm surface and eyes open (H_FSEO_); hip-width, firm surface and eyes closed (H_FSEC_); hip-width, compliant surface and eyes open (H_CSEO_); hip-width, compliant surface and eyes closed (H_CSEC_); tandem, firm surface and eyes open (T_FSEO_); tandem, firm surface and turning the head (T_FSTH_); tandem, compliant surface and eyes open (T_CSEO_).  Four comparisons; Bonferroni-adjusted statistical significance level for correlation: **P*<.013. | | | | | | | | | |

| **Table S7.**  Relationship between assessment methods of trunk stability and whole-body balance with assessment methods of straight-line gait capability and the capability to move by making a change of direction. | | | | | | | | | | | | |
| --- | --- | --- | --- | --- | --- | --- | --- | --- | --- | --- | --- | --- |
|  | | | |  | | **Gait-related tests** | | | | | | |
|  | | | | | **Straight-line gait tests** | | | | | | | **Functional mobility test** |
|  | |  |  | | **Periodicity index** (%) | | | **Walking speed** (m/s) | | | |  |
|  | |  |  | | G_LPI_ | | G_FPI_ | G_HFWS_ | G_THWS_ | G_LWS_ | G_FWS_ | **Modified Timed Up & Go test** (s) |
|  | **Unstable sitting posturographic test** (mm) | | | |  | |  |  |  |  |  |  |
|  |  |  |  |  | -0.055 | | -0.184 | -0.035 | -0.078 | -0.054 | 0.014 | 0.294 |
|  | **Lumbopelvic stability tests** (m/s^2^) | | | |  | |  |  |  |  |  |  |
| **Trunk stability tests** |  | Back bridge with single-leg support | BB_SL_ | | -0.208 | | -0.324 | 0.079 | -0.080 | 0.173 | 0.074 | -0.234 |
|  |  | Back bridge with single-leg support on a hemisphere ball | BB_SLB_ | | -0.270 | | -0.400 | -0.087 | -0.248 | 0.098 | 0.163 | -0.174 |
|  |  | Side bridge with short single-leg support | SB_SSL_ | | -0.370 | | -0.191 | 0.078 | -0.017 | 0.003 | 0.071 | -0.015 |
|  |  | Side bridge with long double-leg support | SB_LDL_ | | -0.564 | | 0.000 | 0.139 | -0.243 | 0.139 | 0.094 | 0.014 |
|  |  | Front bridge with short double-leg support and arms on hemisphere ball | FB_SDLB_ | | -0.377 | | -0.219 | 0.020 | -0.068 | 0.013 | 0.065 | 0.016 |
|  |  | Front bridge with long double-leg support | FB_LDL_ | | -0.228 | | -0.014 | 0.117 | 0.030 | 0.167 | 0.158 | -0.047 |
|  |  | Front bridge with long single-leg support | FB_LSL_ | | -0.145 | | -0.004 | -0.215 | 0.007 | 0.267 | 0.313 | -0.169 |
|  |  | Two-limb bird-dog position | BD_2L_ | | -0.323 | | -0.273 | -0.155 | -0.049 | 0.205 | 0.152 | 0.078 |
| **Whole-body balance tests** | **Whole-body dynamic balance test** | **Tandem stance posturographic test** (mm) |  | |  | |  |  |  |  |  |  |
|  |  |  |  | | 0.138 | | -0.007 | 0.155 | 0.117 | 0.122 | 0.068 | 0.280 |
|  | **Whole-body static**  **balance tests** | **Tests with feet positioned hip-width apart** (m/s^2^) |  | |  | |  |  |  |  |  |  |
|  |  | Hip-width, firm surface and eyes open | H_FSEO_ | | 0.010 | | -0.289 | 0.399* | 0.205 | 0.338 | 0.315 | -0.444* |
|  |  | Hip-width, firm surface and eyes closed | H_FSEC_ | | -0.015 | | -0.331 | 0.393* | 0.210 | 0.379* | 0.351 | -0.489* |
|  |  | Hip-width, compliant surface and eyes open | H_CSEO_ | | 0.005 | | -0.236 | 0.377* | 0.216 | 0.407* | 0.336 | -0.453* |
|  |  | Hip-width, compliant surface and eyes closed | H_CSEC_ | | -0.056 | | -0.205 | 0.248 | 0.083 | 0.328 | 0.287 | -0.501* |
|  |  | **Tests with feet positioned in tandem** (m/s^2^) |  | |  | |  |  |  |  |  |  |
|  |  | Tandem, firm surface and eyes open | T_FSEO_ | | -0.201 | | -0.142 | 0.114 | -0.063 | 0.088 | 0.027 | -0.075 |
|  |  | Tandem, firm surface and turning the head | T_FSTH_ | | -0.218 | | -0.202 | 0.152 | 0.049 | 0.100 | 0.035 | -0.095 |
|  |  | Tandem, compliant surface and eyes open | T_CSEO_ | | 0.003 | | -0.065 | 0.103 | -0.021 | 0.054 | 0.085 | -0.055 |
| Straight-line gait tests: one-sided 10% body weight load gait periodicity index (G_LPI_); fast gait without running periodicity index (G_FPI_); head-forward gait walking speed (G_HFWS_); head turning gait walking speed (G_HTWS_); one-sided 10% body weight load gait walking speed (G_LWS_); fast gait without running walking speed (G_FWS_).  Eight comparisons; Bonferroni-adjusted statistical significance level for correlation: **P*<.006. | | | | | | | | | | | | |

| **Table S8.** Sensitivity analyses of absolute and relative between-session reliability for the trunk stability tests using mean performance–based data reduction. | | | | | | | | | | | | | | | | | | | |
| --- | --- | --- | --- | --- | --- | --- | --- | --- | --- | --- | --- | --- | --- | --- | --- | --- | --- | --- | --- |
|  | |  | n | **Session 1 (mean ± SD)** | | | **Session 2 (mean ± SD)** | | | **Typical error** | | | | | **ICC Mean (LCL - UCL)** | | | |  |
|  |  |  |  |  |  |  |  |  |  | **Mean (LCL - UCL)** | | | | **%** |  |  |  |  |  |
| **Unstable sitting** **posturographic test** (mm) | |  | 53 | 20.56 | ± | 6.74 | 16.18 | ± | 6.45 | 3.33 | 2.80 | - | 4.12 | 17.84 | 0.75 | 0.61 | - | 0.85 |  |
| **Lumbopelvic stability tests** (m/s^2^) | Back bridge with double-leg support | BB_DL_ | 51 | 0.17 | ± | 0.06 | 0.15 | ± | 0.06 | 0.06 | 0.05 | - | 0.07 | 34.73 | 0.22 | -0.06 | - | 0.46 |  |
|  | Back bridge with single-leg support | BB_SL_ | 43 | 0.25 | ± | 0.11 | 0.24 | ± | 0.09 | 0.04 | 0.03 | - | 0.05 | 17.36 | 0.84 | 0.72 | - | 0.91 |  |
|  | Back bridge with single-leg support on a hemisphere ball | BB_SLB_ | 29 | 0.28 | ± | 0.10 | 0.27 | ± | 0.10 | 0.04 | 0.03 | - | 0.06 | 15.21 | 0.84 | 0.69 | - | 0.92 |  |
|  | Side bridge with short double-leg support | SB_SDL_ | 46 | 0.23 | ± | 0.07 | 0.21 | ± | 0.08 | 0.05 | 0.04 | - | 0.07 | 24.98 | 0.51 | 0.26 | - | 0.70 |  |
|  | Side bridge with short single-leg support | SB_SSL_ | 39 | 0.21 | ± | 0.08 | 0.20 | ± | 0.08 | 0.03 | 0.03 | - | 0.04 | 15.53 | 0.83 | 0.70 | - | 0.91 |  |
|  | Side bridge with long double-leg support | SB_LDL_ | 20 | 0.34 | ± | 0.13 | 0.31 | ± | 0.10 | 0.05 | 0.04 | - | 0.07 | 14.45 | 0.85 | 0.66 | - | 0.94 |  |
|  | Front bridge with short double-leg support and arms on a hemisphere ball | FB_SDLB_ | 52 | 0.11 | ± | 0.05 | 0.12 | ± | 0.08 | 0.06 | 0.05 | - | 0.07 | 47.79 | 0.36 | 0.10 | - | 0.58 |  |
|  | Front bridge with long double-leg support | FB_LDL_ | 51 | 0.24 | ± | 0.09 | 0.23 | ± | 0.08 | 0.03 | 0.03 | - | 0.04 | 13.21 | 0.88 | 0.79 | - | 0.93 |  |
|  | Front bridge with long single-leg support | FB_LSL_ | 34 | 0.37 | ± | 0.14 | 0.35 | ± | 0.11 | 0.06 | 0.05 | - | 0.08 | 16.22 | 0.79 | 0.62 | - | 0.89 |  |
|  | Three-limb bird-dog position | BD_3L_ | 53 | 0.14 | ± | 0.06 | 0.14 | ± | 0.05 | 0.04 | 0.03 | - | 0.05 | 28.91 | 0.41 | 0.16 | - | 0.61 |  |
|  | Two-limb bird-dog position | BD_2L_ | 50 | 0.21 | ± | 0.06 | 0.21 | ± | 0.07 | 0.03 | 0.02 | - | 0.03 | 12.47 | 0.84 | 0.74 | - | 0.91 |  |
|  | Two-limb bird-dog position with the forefoot of the supporting leg elevated | BD_2LF_ | 15 | 0.64 | ± | 0.21 | 0.57 | ± | 0.24 | 0.17 | 0.12 | - | 0.26 | 27.62 | 0.47 | -0.04 | - | 0.78 |  |
| SD: standard deviation; ICC: intraclass correlation coefficient; LCL: lower confidence limit set at 95%; UCL: upper confidence limit set at 95%. | | | | | | | | | | | | | | | | | | | |

| **Table S9.** Sensitivity analyses of absolute and relative between-session reliability for the whole-body balance, gait, and functional mobility tests using mean performance–based data reduction. | | | | | | | | | | | | | | | | | | | |  |
| --- | --- | --- | --- | --- | --- | --- | --- | --- | --- | --- | --- | --- | --- | --- | --- | --- | --- | --- | --- | --- |
|  | | |  | n | **Session 1 (mean ± SD)** | | | **Session 2 (mean ± SD)** | | | **Typical error** | | | | | **ICC Mean (LCL - UCL)** | | | | |
|  |  |  |  |  |  |  |  |  |  |  | **Mean (LCL - UCL)** | | | | **%** |  |  |  |  |  |
| **Whole-body dynamic balance test** | **Tandem stance posturographic test** (mm) | |  | 53 | 16.70 | ± | 7.34 | 14.19 | ± | 4.71 | 4.47 | 3.75 | - | 5.53 | 28.94 | 0.48 | 0.25 | - | 0.66 | |
| **Whole-body static balance tests** | **Tests with feet positioned hip-width apart** (m/s^2^) | Hip-width, firm surface and eyes open | H_FSEO_ | 51 | 0.033 | ± | 0.012 | 0.032 | ± | 0.009 | 0.004 | 0.004 | - | 0.005 | 13.58 | 0.83 | 0.73 | - | 0.90 | |
|  |  | Hip-width, firm surface and eyes closed | H_FSEC_ | 51 | 0.036 | ± | 0.014 | 0.035 | ± | 0.014 | 0.006 | 0.005 | - | 0.007 | 16.58 | 0.83 | 0.72 | - | 0.90 | |
|  |  | Hip-width, compliant surface and eyes open | H_CSEO_ | 51 | 0.043 | ± | 0.016 | 0.041 | ± | 0.014 | 0.008 | 0.007 | - | 0.010 | 18.91 | 0.73 | 0.56 | - | 0.83 | |
|  |  | Hip-width, compliant surface and eyes closed | H_CSEC_ | 51 | 0.052 | ± | 0.024 | 0.049 | ± | 0.020 | 0.012 | 0.010 | - | 0.015 | 24.57 | 0.69 | 0.51 | - | 0.81 | |
|  | **Tests with feet positioned in tandem** (m/s^2^) | Tandem, firm surface and eyes open | T_FSEO_ | 47 | 0.11 | ± | 0.04 | 0.12 | ± | 0.06 | 0.03 | 0.03 | - | 0.04 | 26.37 | 0.62 | 0.41 | - | 0.77 | |
|  |  | Tandem, firm surface and turning the head | T_FSTH_ | 45 | 0.17 | ± | 0.07 | 0.19 | ± | 0.08 | 0.04 | 0.03 | - | 0.05 | 21.56 | 0.74 | 0.58 | - | 0.85 | |
|  |  | Tandem, compliant surface and eyes open | T_CSEO_ | 43 | 0.18 | ± | 0.09 | 0.18 | ± | 0.07 | 0.05 | 0.04 | - | 0.06 | 27.69 | 0.59 | 0.35 | - | 0.75 | |
|  |  | Tandem, firm surface and eyes closed | T_FSEC_ | 27 | 0.19 | ± | 0.07 | 0.20 | ± | 0.08 | 0.06 | 0.04 | - | 0.08 | 29.03 | 0.46 | 0.10 | - | 0.71 | |
| **Straight-line gait tests** | **Periodicity index** (%) | Head-forward gait periodicity index | G_HFPI_ | 45 | 68.88 | ± | 3.01 | 69.12 | ± | 3.29 | 2.59 | 2.15 | - | 3.28 | 3.76 | 0.33 | 0.04 | - | 0.57 | |
|  |  | Head turning gait periodicity index | G_HTPI_ | 48 | 65.69 | ± | 4.05 | 66.48 | ± | 4.99 | 3.60 | 3.00 | - | 4.51 | 5.45 | 0.38 | 0.11 | - | 0.60 | |
|  |  | One-sided 10% body weight load gait periodicity index | G_LPI_ | 44 | 67.96 | ± | 2.95 | 67.75 | ± | 3.54 | 2.23 | 1.48 | - | 2.82 | 3.28 | 0.54 | 0.30 | - | 0.72 | |
|  |  | Fast gait without running periodicity index | G_FPI_ | 45 | 68.93 | ± | 365 | 67.47 | ± | 5.59 | 3.26 | 2.70 | - | 4.12 | 4.79 | 0.53 | 0.28 | - | 0.71 | |
|  | **Walking speed** (m/s) | Head-forward gait walking speed | G_HFWS_ | 50 | 1.27 | ± | 0.19 | 1.36 | ± | 0.20 | 0.12 | 0.10 | - | 0.16 | 9.47 | 0.61 | 0.40 | - | 0.76 | |
|  |  | Head turning gait walking speed | G_HTWS_ | 50 | 1.12 | ± | 0.24 | 1.25 | ± | 0.22 | 0.14 | 0.12 | - | 0.18 | 11.90 | 0.63 | 0.43 | - | 0.77 | |
|  |  | One-sided 10% body weight load gait walking speed | G_LWS_ | 50 | 1.33 | ± | 0.19 | 1.41 | ± | 0.22 | 0.09 | 0.08 | - | 0.12 | 6.85 | 0.79 | 0.66 | - | 0.88 | |
|  |  | Fast gait without running walking speed | G_FWS_ | 50 | 1.64 | ± | 0.25 | 1.69 | ± | 0.29 | 0.13 | 0.11 | - | 0.16 | 7.59 | 0.78 | 0.65 | - | 0.87 | |
| **Functional mobility test** | **Modified Timed Up & Go test** (s) | |  | 53 | 6.09 | ± | 1.10 | 5.70 | ± | 1.01 | 0.35 | 0.29 | - | 0.43 | 5.90 | 0.90 | 0.83 | - | 0.94 | |
| SD: standard deviation; ICC: intraclass correlation coefficient; LCL: lower confidence limit set at 95%; UCL: upper confidence limit set at 95%. | | | | | | | | | | | | | | | | | | | |  |

| **Table S10.** Sensitivity analyses of the relationships between laboratory- and field-based trunk stability measures using the average of Session 1 and Session 2 mean performance. | | | | | | | | | |
| --- | --- | --- | --- | --- | --- | --- | --- | --- | --- |
|  |  | BB_SL_ | BB_SLB_ | SB_SSL_ | SB_LDL_ | FB_SDLB_ | FB_LDL_ | FB_LSL_ | BD_2L_ |
| **Unstable sitting posturographic test** (mm) |  |  |  |  |  |  |  |  |  |
|  |  | -0.092 | 0.066 | 0.222 | 0.174 | 0.245 | 0.182 | 0.362* | 0.403* |
| **Lumbopelvic stability tests** (m/s^2^) |  |  |  |  |  |  |  |  |  |
| Back bridge with single-leg support | BB_SL_ | - | 0.794* | 0.521* | 0.383 | 0.279 | 0.284 | 0.372* | 0.532* |
| Back bridge with single-leg support on a hemisphere ball | BB_SLB_ |  | - | 0.616* | 0.415 | 0.511* | 0.444* | 0.587* | 0.621* |
| Side bridge with short single-leg support | SB_SSL_ |  |  | - | 0.765* | 0.414* | 0.631* | 0.634* | 0.518* |
| Side bridge with long double-leg support | SB_LDL_ |  |  |  | - | 0.510* | 0.759* | 0.511* | 0.563* |
| Front bridge with short double-leg support and arms on hemisphere ball | FB_SDLB_ |  |  |  |  | - | 0.440* | 0.446* | 0.444* |
| Front bridge with long double-leg support | FB_LDL_ |  |  |  |  |  | - | 0.621* | 0.428* |
| Front bridge with long single-leg support | FB_LSL_ |  |  |  |  |  |  | - | 0.652* |
| Two-limb bird-dog position | BD_2L_ |  |  |  |  |  |  |  | - |
| One comparison; statistical significance level for correlation: * *p*<.05. | | | | | | | | | |

| **Table S11.** Sensitivity analyses of the relationships between the two methodologies to evaluate whole-body balance using the average of Session 1 and Session 2 mean performance. | | | | | | | | | | | |
| --- | --- | --- | --- | --- | --- | --- | --- | --- | --- | --- | --- |
|  | | | | | **Whole-body static balance tests** | | | | | | |
|  |  |  |  |  | **Tests with feet positioned**  **hip-width apart** (m/s^2^) | | | | **Tests with feet positioned in tandem** (m/s^2^) | | |
|  |  | | |  | H_FSEO_ | H_FSEC_ | H_CSEO_ | H_CSEC_ | T_FSEO_ | T_FSTH_ | T_CSEO_ |
| **Whole-body dynamic balance test** | **Tandem stance posturographic test** (mm) | | |  |  |  |  |  |  |  |  |
|  |  |  |  |  | 0.005 | -0.066 | 0.077 | -0.001 | 0.437* | 0.447* | 0.403* |
| **Whole-body static balance tests** | **Tests with feet positioned hip-width apart** (m/s^2^) | | | |  |  |  |  |  |  |  |
|  | Hip-width, firm surface and eyes open | | | H_FSEO_ | - | 0.936* | 0.815* | 0.700* | 0.515* | 0.569* | 0.342* |
|  | Hip-width, firm surface and eyes closed | | | H_FSEC_ |  | - | 0.822* | 0.776* | 0.444* | 0.447* | 0.323* |
|  | Hip-width, compliant surface and eyes open | | | H_CSEO_ |  |  | - | 0.901* | 0.593* | 0.576* | 0.520* |
|  | Hip-width, compliant surface and eyes closed | | | H_CSEC_ |  |  |  | - | 0.454* | 0.370* | 0.508* |
|  | **Tests with feet positioned in tandem** (m/s^2^) | | |  |  |  |  |  |  |  |  |
|  | Tandem, firm surface and eyes open | | | T_FSEO_ |  |  |  |  | - | 0.829* | 0.766* |
|  | Tandem, firm surface and turning the head | | | T_FSTH_ |  |  |  |  |  | - | 0.695* |
|  | Tandem, compliant surface and eyes open | | | T_CSEO_ |  |  |  |  |  |  | - |
| One comparison; statistical significance level for correlation: * *p*<.05. | | | | | | | | | | | |

| **Table S12.** Sensitivity analyses of the relationships between the two assessment methods representing trunk stability and the two representing whole-body balance using the average of Session 1 and Session 2 mean performance. | | | | | | | | | |
| --- | --- | --- | --- | --- | --- | --- | --- | --- | --- |
|  |  | **Whole-body dynamic balance test** | **Whole-body static balance tests** | | | | | | |
|  |  |  | **Tests with feet positioned hip-width apart** (m/s^2^) | | | | **Tests with feet positioned in tandem** (m/s^2^) | | |
|  |  | **Tandem stance posturographic test** (mm) | H_FSEO_ | H_FSEC_ | H_CSEO_ | H_CSEC_ | T_FSEO_ | T_FSTH_ | T_CSEO_ |
| **Unstable sitting posturographic test** (mm) | |  |  |  |  |  |  |  |  |
|  |  | 0.527* | 0.025 | -0.065 | 0.001 | 0.005 | 0.252 | 0.228 | 0.271 |
| **Lumbopelvic stability tests** (m/s^2^) | |  |  |  |  |  |  |  |  |
|  | BB_SL_ | -0.002 | 0.618* | 0.624* | 0.532* | 0.496* | 0.274 | 0.310 | 0.273 |
|  | BB_SLB_ | -0.029 | 0.659* | 0.592* | 0.472 | 0.430 | 0.406 | 0.682* | 0.586* |
|  | SB_SSL_ | 0.145 | 0.357 | 0.438* | 0.145 | 0.224 | 0.252 | 0.148 | 0.351 |
|  | SB_LDL_ | -0.044 | 0.333 | 0.286 | 0.069 | 0.156 | 0.556 | 0.415 | 0.054 |
|  | FB_SDLB_ | 0.002 | 0.328 | 0.224 | 0.193 | 0.054 | 0.264 | 0.440* | 0.255 |
|  | FB_LDL_ | 0.165 | 0.267 | 0.271 | 0.190 | 0.223 | 0.504* | 0.455* | 0.380* |
|  | FB_LSL_ | 0.202 | 0.286 | 0.262 | 0.271 | 0.347 | 0.492* | 0.470* | 0.585* |
|  | BD_2L_ | 0.398* | 0.460* | 0.378* | 0.465* | 0.387* | 0.547* | 0.519* | 0.526* |
| The lumbopelvic stability test positions: back bridge with single-leg support (BB_SL_); back bridge with single-leg support on a hemisphere ball (BB_SLB_); side bridge with short single-leg support (SB_SSL_); side bridge with long double-leg support (SB_LDL_); front bridge with short double-leg support and arms on hemisphere ball (FB_SDLB_); front bridge with long double-leg support (FB_LDL_); front bridge with long single-leg support (FB_LSL_); two-limb bird-dog position (BD_2L_). Whole-body static balance tests: hip-width, firm surface and eyes open (H_FSEO_); hip-width, firm surface and eyes closed (H_FSEC_); hip-width, compliant surface and eyes open (H_CSEO_); hip-width, compliant surface and eyes closed (H_CSEC_); tandem, firm surface and eyes open (T_FSEO_); tandem, firm surface and turning the head (T_FSTH_); tandem, compliant surface and eyes open (T_CSEO_).  Four comparisons; Bonferroni-adjusted statistical significance level for correlation: **p*<.013. | | | | | | | | | |

| **Table S13.** Sensitivity analyses of the relationships between assessment methods of trunk stability and whole-body balance with assessment methods of straight-line gait capability and the capability to move by making a change of direction using the average of Session 1 and Session 2 mean performance. | | | | | | | | | | | | |
| --- | --- | --- | --- | --- | --- | --- | --- | --- | --- | --- | --- | --- |
|  | | | |  | | **Gait-related tests** | | | | | | |
|  | | | | | **Straight-line gait tests** | | | | | | | **Functional mobility test** |
|  | |  |  | | **Periodicity index** (%) | | | **Walking speed** (m/s) | | | |  |
|  | |  |  | | G_LPI_ | | G_FPI_ | G_HFWS_ | G_THWS_ | G_LWS_ | G_FWS_ | **Modified Timed Up & Go test** (s) |
|  | **Unstable sitting posturographic test** (mm) | | | |  | |  |  |  |  |  |  |
|  |  |  |  |  | -0.126 | | -0.151 | -0.120 | -0.210 | -0.019 | -0.145 | 0.414* |
|  | **Lumbopelvic stability tests** (m/s^2^) | | | |  | |  |  |  |  |  |  |
| **Trunk stability tests** |  | Back bridge with single-leg support | BB_SL_ | | -0.195 | | -0.265 | -0.008 | 0.008 | 0.131 | 0.043 | -0.197 |
|  |  | Back bridge with single-leg support on a hemisphere ball | BB_SLB_ | | -0.317 | | -0.150 | 0.062 | -0.079 | 0.030 | 0.139 | -0.295 |
|  |  | Side bridge with short single-leg support | SB_SSL_ | | -0.340 | | -0.148 | 0.002 | -0.097 | 0.015 | 0.078 | 0.024 |
|  |  | Side bridge with long double-leg support | SB_LDL_ | | -0.637 | | -0.182 | 0.015 | -0.197 | 0.046 | 0.079 | -0.270 |
|  |  | Front bridge with short double-leg support and arms on hemisphere ball | FB_SDLB_ | | -0.116 | | -0.221 | 0.208 | 0.174 | 0.129 | 0.274 | -0.134 |
|  |  | Front bridge with long double-leg support | FB_LDL_ | | -0.322 | | -0.097 | 0.163 | 0.081 | 0.112 | 0.159 | 0.069 |
|  |  | Front bridge with long single-leg support | FB_LSL_ | | -0.161 | | 0.120 | 0.223 | 0.052 | 0.201 | 0.267 | -0.035 |
|  |  | Two-limb bird-dog position | BD_2L_ | | -0.300 | | -0.235 | 0.300 | 0.060 | 0.195 | 0.216 | 0.122 |
| **Whole-body balance tests** | **Whole-body dynamic balance test** | **Tandem stance posturographic test** (mm) |  | |  | |  |  |  |  |  |  |
|  |  |  |  | | -0.168 | | -0.010 | -0.004 | -0.080 | -0.121 | -0.163 | 0.423* |
|  | **Whole-body static**  **balance tests** | **Tests with feet positioned hip-width apart** (m/s^2^) |  | |  | |  |  |  |  |  |  |
|  |  | Hip-width, firm surface and eyes open | H_FSEO_ | | -0.062 | | -0.408 | 0.434* | 0.281 | 0.410* | 0.327 | -0.390* |
|  |  | Hip-width, firm surface and eyes closed | H_FSEC_ | | -0.029 | | -0.378 | 0.334 | 0.229 | 0.364 | 0.335 | -0.401* |
|  |  | Hip-width, compliant surface and eyes open | H_CSEO_ | | -0.074 | | -0.402 | 0.421* | 0.348 | 0.409* | 0.313 | -0.387* |
|  |  | Hip-width, compliant surface and eyes closed | H_CSEC_ | | -0.105 | | -0.369 | 0.317 | 0.239 | 0.387 | 0.339 | -0.426* |
|  |  | **Tests with feet positioned in tandem** (m/s^2^) |  | |  | |  |  |  |  |  |  |
|  |  | Tandem, firm surface and eyes open | T_FSEO_ | | -0.234 | | -0.240 | 0.243 | 0.110 | 0.033 | 0.016 | 0.012 |
|  |  | Tandem, firm surface and turning the head | T_FSTH_ | | -0.188 | | -0.216 | 0.279 | 0.192 | 0.050 | 0.134 | -0.123 |
|  |  | Tandem, compliant surface and eyes open | T_CSEO_ | | -0.048 | | -0.112 | 0.206 | 0.103 | 0.004 | 0.079 | 0.024 |
| Straight-line gait tests: one-sided 10% body weight load gait periodicity index (G_LPI_); fast gait without running periodicity index (G_FPI_); head-forward gait walking speed (G_HFWS_); head turning gait walking speed (G_HTWS_); one-sided 10% body weight load gait walking speed (G_LWS_); fast gait without running walking speed (G_FWS_).  Eight comparisons; Bonferroni-adjusted statistical significance level for correlation: **p*<.006. | | | | | | | | | | | | |

| 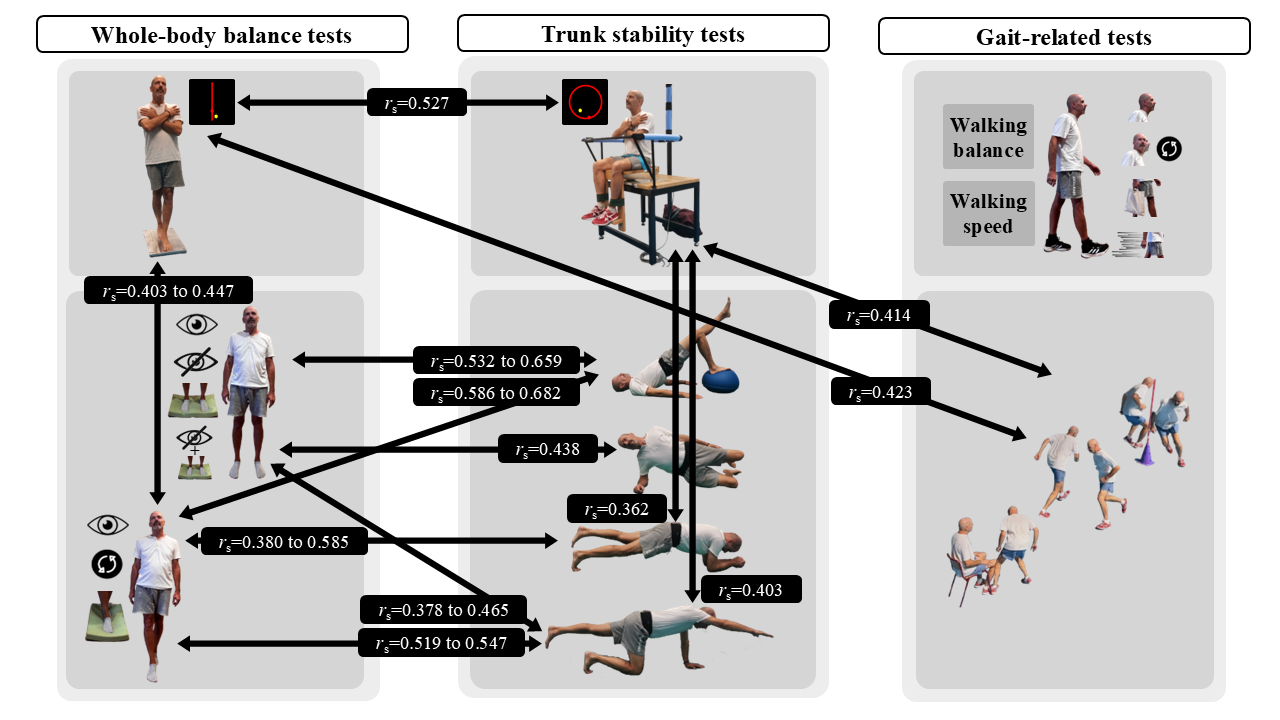 |
| --- |
| **Figure S1.** Sensitivity analyses of the correlations between trunk stability, whole-body balance, and gait-related outcomes using mean performance averaged across Sessions 1 and 2. The overall pattern of associations remained consistent with the primary analysis based on Session 2 peak performance, with predominantly low, selective, and task-specific relationships. A limited number of correlations increased from non-significant or low to low–moderate and reached statistical significance, particularly between the unstable sitting posturographic test and selected lumbopelvic stability tasks (front bridge with long single-leg support and two-limb bird-dog), as well as between the unstable sitting and tandem stance dynamic posturographic tests and the modified Timed Up & Go test. These modest increases are interpreted as the expected consequence of using more stable performance estimates, reflecting reduced random measurement error and day-to-day variability, without altering the qualitative interpretation of the findings. |

| **Table S14.**  Focused sensitivity analyses of descriptive statistics and absolute and relative between-session reliability for the lumbopelvic stability tests restricted to participants who successfully completed all three difficulty levels. | | | | | | | | | | | | | | | | | | | |
| --- | --- | --- | --- | --- | --- | --- | --- | --- | --- | --- | --- | --- | --- | --- | --- | --- | --- | --- | --- |
|  | |  | n | **Session 1 (mean ± SD)** | | | **Session 2 (mean ± SD)** | | | **Typical error** | | | | | **ICC Mean (LCL - UCL)** | | | |  |
|  |  |  |  |  |  |  |  |  |  | **Mean (LCL - UCL)** | | | | **%** |  |  |  |  |  |
| **Lumbopelvic stability tests** (m/s^2^) | Back bridge with double-leg support | BB_DL_ | 29 | 0.16 | ± | 0.06 | 0.15 | ± | 0.07 | 0.06 | 0.05 | - | 0.09 | 41.16 | 0.13 | -0.25 | - | 0.47 |  |
|  | Back bridge with single-leg support | BB_SL_ | 29 | 0.23 | ± | 0.13 | 0.22 | ± | 0.09 | 0.05 | 0.04 | - | 0.07 | 21.81 | 0.82 | 0.65 | - | 0.91 |  |
|  | Back bridge with single-leg support on a hemisphere ball | BB_SLB_ | 29 | 0.25 | ± | 0.09 | 0.24 | ± | 0.09 | 0.04 | 0.03 | - | 0.05 | 16.20 | 0.83 | 0.66 | - | 0.91 |  |
|  | Side bridge with short double-leg support | SB_SDL_ | 18 | 0.18 | ± | 0.07 | 0.17 | ± | 0.05 | 0.05 | 0.04 | - | 0.07 | 28.53 | 0.32 | -0.16 | - | 0.68 |  |
|  | Side bridge with short single-leg support | SB_SSL_ | 18 | 0.19 | ± | 0.08 | 0.18 | ± | 0.07 | 0.02 | 0.02 | - | 0.03 | 11.61 | 0.93 | 0.83 | - | 0.97 |  |
|  | Side bridge with long double-leg support | SB_LDL_ | 18 | 0.30 | ± | 0.09 | 0.27 | ± | 0.07 | 0.04 | 0.03 | - | 0.06 | 14.09 | 0.79 | 0.52 | - | 0.91 |  |
|  | Front bridge with short double-leg support and arms on a hemisphere ball | FB_SDLB_ | 34 | 0.09 | ± | 0.05 | 0.10 | ± | 0.07 | 0.03 | 0.03 | - | 0.04 | 34.34 | 0.70 | 0.47 | - | 0.84 |  |
|  | Front bridge with long double-leg support | FB_LDL_ | 34 | 0.20 | ± | 0.09 | 0.20 | ± | 0.07 | 0.03 | 0.03 | - | 0.04 | 15.63 | 0.86 | 0.74 | - | 0.93 |  |
|  | Front bridge with long single-leg support | FB_LSL_ | 34 | 0.33 | ± | 0.11 | 0.32 | ± | 0.10 | 0.04 | 0.03 | - | 0.05 | 12.44 | 0.85 | 0.72 | - | 0.92 |  |
|  | Three-limb bird-dog position | BD_3L_ | 15 | 0.14 | ± | 0.06 | 0.13 | ± | 0.06 | 0.05 | 0.03 | - | 0.07 | 35.00 | 0.33 | -0.20 | - | 0.71 |  |
|  | Two-limb bird-dog position | BD_2L_ | 15 | 0.19 | ± | 0.07 | 0.19 | ± | 0.07 | 0.02 | 0.02 | - | 0.04 | 12.81 | 0.90 | 0.73 | - | 0.97 |  |
|  | Two-limb bird-dog position with the forefoot of the supporting leg elevated | BD_2LF_ | 15 | 0.58 | ± | 0.20 | 0.55 | ± | 0.23 | 0.19 | 0.14 | - | 0.29 | 33.03 | 0.27 | -0.26 | - | 0.68 |  |
| SD: standard deviation; ICC: intraclass correlation coefficient; LCL: lower confidence limit set at 95%; UCL: upper confidence limit set at 95%. | | | | | | | | | | | | | | | | | | | |

| **Table S15.** Focused sensitivity analyses of the relationships between the two assessment methods representing trunk stability and the two representing whole-body balance, restricted to participants who successfully completed all three difficulty levels for each lumbopelvic stability test position. | | | | | | | | | |
| --- | --- | --- | --- | --- | --- | --- | --- | --- | --- |
|  |  | **Whole-body dynamic balance test** | **Whole-body static balance tests** | | | | | | |
|  |  |  | **Tests with feet positioned hip-width apart** (m/s^2^) | | | | **Tests with feet positioned in tandem** (m/s^2^) | | |
|  |  | **Tandem stance posturographic test** (mm) | H_FSEO_ | H_FSEC_ | H_CSEO_ | H_CSEC_ | T_FSEO_ | T_FSTH_ | T_CSEO_ |
| **Unstable sitting posturographic test** (mm) | |  |  |  |  |  |  |  |  |
|  |  | 0.436* | -0.027 | -0.038 | -0.066 | -0.109 | 0.263 | 0.161 | 0.212 |
| **Lumbopelvic stability tests** (m/s^2^) | |  |  |  |  |  |  |  |  |
|  | BB_SL_ | 0.052 | 0.650* | 0.568* | 0.628* | 0.516* | 0.532* | 0.487 | 0.473 |
|  | BB_SLB_ | 0.066 | 0.643* | 0.579* | 0.692* | 0.673* | 0.458* | 0.680* | 0.700* |
|  | SB_SSL_ | -0.150 | 0.479 | 0.374 | 0.150 | 0.315 | 0.532 | 0.382 | 0.496 |
|  | SB_LDL_ | -0.294 | 0.062 | -0.018 | -0.256 | -0.150 | 0.285 | 0.043 | 0.111 |
|  | FB_SDLB_ | 0.158 | 0.336 | 0.258 | 0.299 | 0.273 | 0.489* | 0.392 | 0.343 |
|  | FB_LDL_ | 0.221 | 0.132 | 0.147 | 0.198 | 0.320 | 0.480* | 0.447 | 0.405 |
|  | FB_LSL_ | 0.149 | 0.416 | 0.337 | 0.437 | 0.497* | 0.380 | 0.425 | 0.666* |
|  | BD_2L_ | 0.211 | 0.496 | 0.329 | 0.432 | 0.550 | 0.614 | 0.481 | 0.318 |
| The lumbopelvic stability test positions: back bridge with single-leg support (BB_SL_); back bridge with single-leg support on a hemisphere ball (BB_SLB_); side bridge with short single-leg support (SB_SSL_); side bridge with long double-leg support (SB_LDL_); front bridge with short double-leg support and arms on hemisphere ball (FB_SDLB_); front bridge with long double-leg support (FB_LDL_); front bridge with long single-leg support (FB_LSL_); two-limb bird-dog position (BD_2L_). Whole-body static balance tests: hip-width, firm surface and eyes open (H_FSEO_); hip-width, firm surface and eyes closed (H_FSEC_); hip-width, compliant surface and eyes open (H_CSEO_); hip-width, compliant surface and eyes closed (H_CSEC_); tandem, firm surface and eyes open (T_FSEO_); tandem, firm surface and turning the head (T_FSTH_); tandem, compliant surface and eyes open (T_CSEO_).  Four comparisons; Bonferroni-adjusted statistical significance level for correlation: **p*<.013. | | | | | | | | | |
